# Dopaminergic neurons lacking Caspase-3 avoid apoptosis but undergo necrosis after MPTP treatment inducing a Galectin-3-dependent selective microglial phagocytic response

**DOI:** 10.1038/s41419-024-07014-9

**Published:** 2024-08-27

**Authors:** Juan García-Revilla, Rocío Ruiz, Ana M. Espinosa-Oliva, Marti Santiago, Irene García-Domínguez, Lluís Camprubí-Ferrer, Sara Bachiller, Tomas Deierborg, Bertrand Joseph, Rocío M. de Pablos, José A. Rodríguez-Gómez, José Luis Venero

**Affiliations:** 1 Experimental Neuroinflammation Laboratory, Department of Experimental Medical Science, Lund University, BMC B11, 221 84 Lund, Sweden; 2 Instituto de Biomedicina de Sevilla, IBiS/Hospital Universitario Virgen del Rocío/CSIC/Universidad de Sevilla, Seville, Spain; 3 Departamento de Bioquímica y Biología Molecular, Facultad de Farmacia, Universidad de Sevilla, Seville, Spain; 4 Faculty of Health Sciences, Universidad Loyola Andalucía, Seville, Spain; 5 Department of Medical Biochemistry, Molecular Biology and Immunology, School of Medicine, University of Seville, Seville, Spain; 6 Institute of Environmental Medicine, Toxicology Unit, Karolinska Institutet, Stockholm, Sweden; 7 Center for Neuromusculoskeletal Restorative Medicine, Shui On Centre, Wan Chai, Hong Kong

**Keywords:** Parkinson's disease, Cell death

## Abstract

Parkinson’s Disease (PD) is a progressive neurodegenerative disorder characterized by the loss of dopaminergic neurons in the Substantia nigra *pars compacta* (SNpc). Apoptosis is thought to play a critical role in the progression of PD, and thus understanding the effects of antiapoptotic strategies is crucial for developing potential therapies. In this study, we developed a unique genetic model to selectively delete *Casp3*, the gene encoding the apoptotic protein caspase-3, in dopaminergic neurons (TH-C3KO) and investigated its effects in response to a subacute regime of 1-methyl-4-phenyl-1,2,3,6-tetrahydropyridine (MPTP) administration, which is known to trigger apoptotic loss of SNpc dopaminergic neurons. We found that *Casp3* deletion did not protect the dopaminergic system in the long term. Instead, we observed a switch in the cell death pathway from apoptosis in wild-type mice to necrosis in TH-C3KO mice. Notably, we did not find any evidence of necroptosis in our model or in in vitro experiments using primary dopaminergic cultures exposed to 1-methyl-4-phenylpyridinium in the presence of pan-caspase/caspase-8 inhibitors. Furthermore, we detected an exacerbated microglial response in the ventral mesencephalon of TH-C3KO mice in response to MPTP, which mimicked the microglia neurodegenerative phenotype (MGnD). Under these conditions, it was evident the presence of numerous microglial phagocytic cups wrapping around apparently viable dopaminergic cell bodies that were inherently associated with galectin-3 expression. We provide evidence that microglia exhibit phagocytic activity towards both dead and stressed viable dopaminergic neurons through a galectin-3-dependent mechanism. Overall, our findings suggest that inhibiting apoptosis is not a beneficial strategy for treating PD. Instead, targeting galectin-3 and modulating microglial response may be more promising approaches for slowing PD progression.

## Introduction

Parkinson’s disease (PD) is the second most common neurodegenerative disease, comprised of both familial and idiopathic forms, behind only Alzheimer’s disease (AD). The degeneration of dopaminergic neurons in the Substantia nigra *pars compacta* (SNpc) has long been established as the underlying cause of the motor symptoms associated with PD, including rigidity and akinesia [1, 2]. Both clinical studies and experimental in vivo models have demonstrated the involvement of different forms of programmed cell death in dopaminergic neuron loss (for review, see [3]). Given that disturbance of mitochondrial homeostasis is a key contributor to dopaminergic cell loss [4, 5], it is reasonable to consider apoptosis as a central mechanism in the cell death process occurring in this disease. In the intrinsic and extrinsic apoptotic pathways, caspase-9 and caspase-8, respectively, act as initiator caspases leading to the activation of caspase-3, which serves as the ultimate executor of apoptosis. Different events associated with the molecular apoptotic cascade have been observed in *postmortem* human brain samples of PD patients, including complex I deficiency, reactive oxygen species generation, and oxidative damage to lipids, proteins, and DNA, among others [6]. Additionally, active caspase-8 and caspase-3 have been detected in nigrostriatal dopaminergic neurons in the brains of PD patients [7, 8].

The subacute variant of the 1-methyl-4-phenyl-1,2,3,6-tetrahydropyridine (MPTP) mouse model exhibits hallmark features of PD, including decreased complex I activity, oxidative stress, and apoptotic cell death [9]. In light of these observations, diverse interventions targeting apoptotic signalling pathways, either upstream or downstream of mitochondrial outer membrane permeabilisation (MOMP), have been investigated for their potential to attenuate the loss of dopaminergic neurons in the MPTP mouse model, yielding variable outcomes [10–12]. In the translation to the in vitro 1-methyl-4-phenylpyridinium (MPP^+^) model, considerable effort was directed toward inhibiting caspase activity. However, the use of different approaches based on MPP^+^ and caspase inhibitors at different dosages has made it difficult to arrive at conclusive findings regarding neuroprotection [7, 13–15]. Nowadays, the general consensus is that halting the apoptotic molecular program downstream of MOMP is ineffective in preventing cell death [16]. Hence, MOMP is recognised as the “point of no return” in the process of apoptotic cell death.

One important outcome of caspase-mediated apoptosis is the ability of immune cells to recognise and clear apoptotic corpses, which helps to prevent the activation of an inflammatory response [17, 18]. Unlike apoptosis, non-programmed cell death or necrosis results in cell membrane collapse, causing intracellular molecules to spill over, further acting as damage-associated molecular patterns (DAMPs). These molecules trigger the activation of inflammatory immune cells, leading to a self-perpetuating inflammatory process that can prolong the cell death process and result in chronic inflammation. In the brain, the neuroinflammatory process is governed by microglia, a cell type with a plethora of functions both in physiological and pathological conditions [19]. Furthermore, neuronal cell death can be either a cause or a consequence of microglial activation, both contributing to the neurodegenerative process. The involvement of microglia in the pathogenesis of PD has been demonstrated in both patients and animal disease models. Prove of that is the observed induction of enzymatic systems such as nicotinamide adenine dinucleotide phosphate hydrogen oxidase (NADPH-oxidase) and inducible nitric oxide synthase (iNOS), which produce toxic levels of superoxide (O_2_^-^) and nitric oxide (NO·) free radicals, respectively [20, 21]. Microglia play a crucial role in pathological conditions and can exist in various activating states [22]. To better understand the heterogeneity of microglia subpopulations, high-throughput techniques such as single-cell RNA sequencing (scRNA-seq) and immune profiling are being employed. Using these techniques, two independent studies identified a common activated microglial phenotype, referred to as either disease-associated microglia (DAM) [23] or microglial neurodegenerative phenotype (MGnD) [24]. Notably, while the former has been postulated to have neuroprotective effects, the latter has been associated with neurotoxicity. One of the highly upregulated genes in the MGnD phenotype is *Lgals3*, which codifies the protein galectin-3 (GAL3), a pleiotropic protein that binds to β-galactoside residues present in glycoproteins (for review, see [25]). GAL3 is capable of interacting with various receptors, including toll-like receptors (TLRs), triggering receptor expressed on myeloid cells 2 (TREM2), and MER proto-oncogene tyrosine kinase (MERTK), among others, resulting in a wide range of responses that may have beneficial or detrimental effects depending on the specific brain context. Notably, one crucial aspect of GAL3’s impact on microglia activation is its ability to initiate the phagocytic response, serving as a ligand for MERTK [26] or as an opsonin [27, 28] for both dying and stressed but viable cells.

To gain a better understanding of the mechanisms behind dopaminergic neuron death in PD, we administered a subacute dose regime of the neurotoxin MPTP to a mouse genetic model with a selective deletion of the caspase-3 gene (*Casp3*) in tyrosine hydroxylase (TH) expressing neurons [29]. By using this approach, we were able to study the outcome of blocking apoptotic cell death in dopaminergic neurons. As a result of these interventions, we observed a switch in the cell death mode of these neurons from apoptosis to necrosis. Further in vivo and in vitro studies failed to reveal any sign of necroptosis, a form of programmed cell death [30]. Remarkably, the resulting neuronal necrosis had a dramatic effect on the microglia profile in the SNpc, as evidenced by a notable increase in the phagocytic activity affecting apparently viable dopaminergic neurons. We provide evidence that GAL3 may act as an opsonin in this process as we were able to recapitulate in an in vitro co-culture set up with N27 and BV2 cell lines in vivo observations suggesting phagocytic promotion activity of GAL3 on stressed and necrotic neurons.

## Material and methods

### Mouse genetic model and treatments

**Fig. 1:**
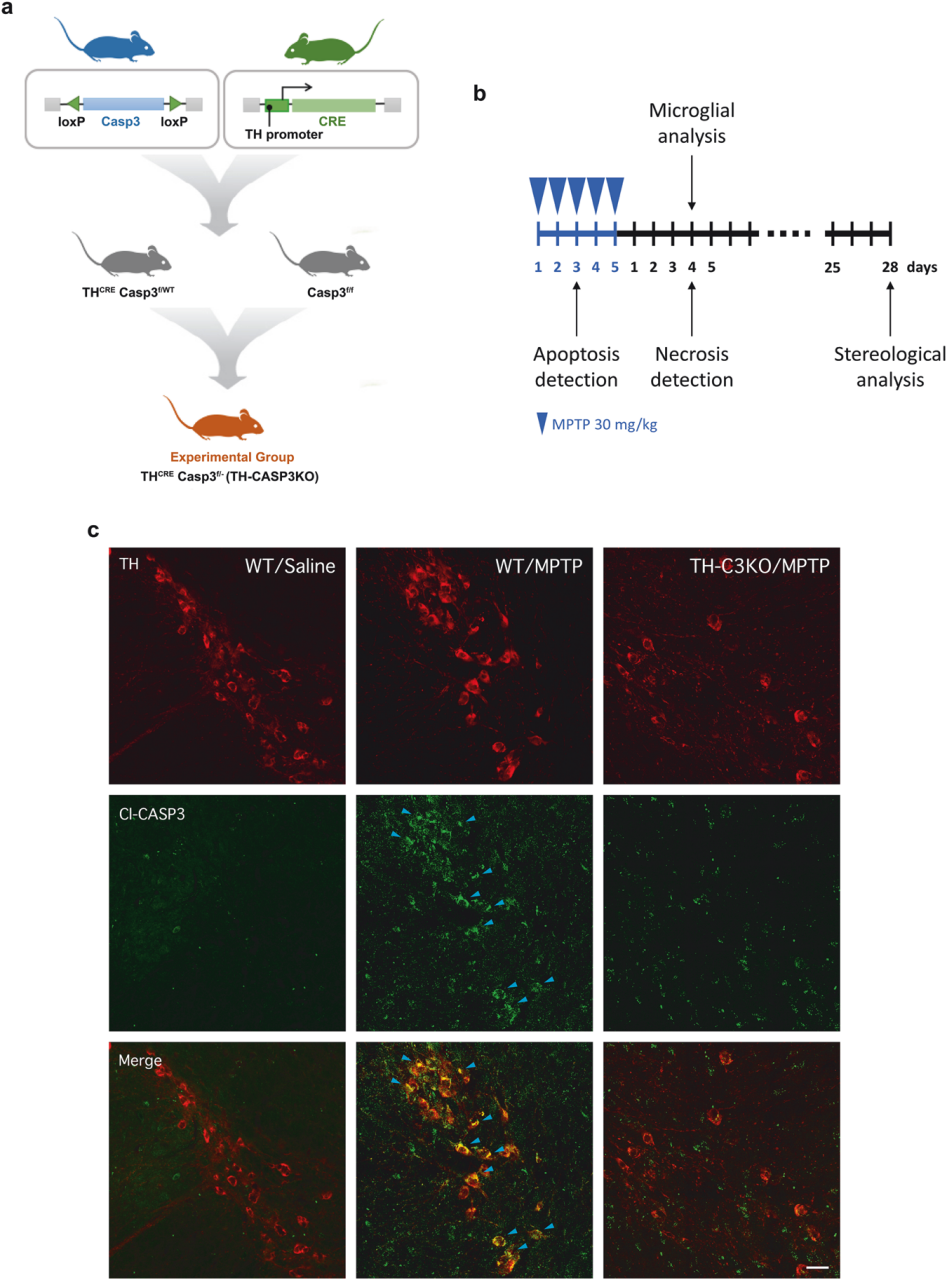
Mouse genetic model, schematic diagram of experiments and proof-of-concept of genetic model. **a** Breeding strategy for the generation of the TH-C3KO mice (experimental group). **b**, Schematic diagram of experiments. WT and TH-C3KO animals received MPTP (30 mg/kg) or saline injections during 5 days and were sacrificed at different time points during and after MPTP treatment. **c** Coronal sections of SNpc immunostained for TH and Cl-CASP3 analyzed at the third day of MPTP injection. Blue arrows in WT/MPTP condition point to TH positive neurons expressing Cl-CASP3. Scale bar, 50 µm.

3 month-old male C57BL/6 wild-type (WT) and the previously generated in our group TH-caspase-3 conditional knockout [29] (hereinafter, TH-C3KO) mice (20–25 g) were obtained from the Center of Animal Experimentation Oscar Pintado (Seville, Spain) (Fig. 1a). Briefly, hemizygous mice carrying *Casp3* deletion were backcrossed with homozygous *Casp3* floxed mice and transgenic mice expressing Cre recombinase enzyme associated to TH promotor. As a result, experimental mice (TH-C3KO) presented one constitutive deleted allele for *Casp3* while the second allele deletion only occurred in the dopaminergic cells. This strategy has proven to be remarkably successful in diminishing caspase-3 expression in dopaminergic neurons [29]. Mice were housed at constant room temperature (RT) of 22 ± 1 °C and relative humidity (60%), with a 12 h light-dark cycle and *ad libitum* access to food and water. Experiments were carried out in accordance with the Guidelines of the European Union Directive (2010/63/EU) and Spanish regulations (BOE 34/11370-421, 2013) for the use of laboratory animals; the study was approved by the Scientific Committee of the University of Seville.

MPTP-HCl (Sigma Aldrich, St. Louis, MO, USA) in 0.9% NaCl was administered intraperitoneally (ip) to WT and TH-C3KO mice, using a subacute dosage of 30 mg/kg/day for five consecutive days. This dosing regimen was selected for its ability to induce apoptotic cell death [31]. The treatment was administered in a volume of 100 µl per 25 g of body weight. Control animals were injected with equal volumes of saline as in the MPTP-treated mice. Animals were sacrificed at different time points depending on the purpose (Fig. 1b). For the study of apoptotic cell death, animals were sacrificed hours after the third, fourth or fifth day of MPTP administration. For the study of necrotic cell death and the analysis of the microglial profile, animals were sacrificed 4 days after completing the MPTP treatment. For long-term histological studies in the SNpc and striatum, animals were sacrificed 28 days after the end of the treatment. At least four animals per group were used in all the experiments.

### Immunohistochemistry

Immunohistochemical detection of TH was performed in the SNpc and processed for stereological analysis. Animals were sacrificed on day 28 after completing MPTP treatment. Mice were transcardially perfused under deep anaesthesia (isoflurane) with 150–200 ml of phosphate-buffered saline (PBS, Nzytech, Lisbon, Portugal), pH 7.4. After perfusion, brains were removed, fixed overnight in 4% paraformaldehyde (PFA, Sigma Aldrich) in PBS, cryoprotected in 30% sucrose in PBS until sunk (2–5 days), and finally frozen in isopentane at −80 °C. Coronal sections 30 µm thick were cut with a cryostat (Leica, Wetzlar, Germany). The following steps were performed at RT unless otherwise noted. Sections were first treated with PBS containing 0.3% hydrogen peroxide (30 min) for inactivation of endogenous peroxidase, then permeabilised with 1% Triton X-100 (Sigma Aldrich) in PBS for 1 h, and later blocked in 5% (w/v) bovine serum albumin (BSA, Sigma Aldrich) in PBS for 1 h before the incubation with a rabbit-derived anti-TH antibody (1:2000, Sigma Aldrich) at 4 °C for 24 h. Subsequently, the sections were incubated with a biotinylated goat anti-rabbit antibody (1:200, Vector laboratories, Newark, CA, USA) for 1.5 h, and later with avidin-biotin complex (ABC, Vector laboratories) conjugated with horseradish peroxidase (1:500) for 1 h. The immunostaining was visualised using the 3,3’-diaminobenzidine tetrahydrochloride (DAB, Sigma Aldrich) reaction (1 min in 0.2 M Tris buffer containing 0.1% DAB and 0.001% hydrogen peroxide). Sections were mounted on gelatin-coated slides, cleared in xylene, and coverslipped using a DPX mounting medium (Sigma Aldrich).

Stereology was made in a manually bounded region of the SNpc based on TH reactivity and with the help of a mouse brain atlas [32]. In each case, we used 6-8 sections per animal, systematically distributed through the anterior-posterior axis of the analysed region. The number of TH^+^ neurons in the SNpc was estimated using a fractionator sampling design [33]. Counts were made at regular predetermined intervals (x = 150 µm and y = 200 µm) within each section. An unbiased counting frame of known area (80 µm × 50 µm = 4000 µm^2^) was superimposed on the tissue section image under a 100x oil immersion objective in an OLYMPUS BX61 motorised microscope (Olympus, Tokyo, Japan). Therefore, the area sampling fraction was 4000 µm^2^/ (150 µm × 200 µm) = 0.133. The entire z-dimension of each section was sampled; hence, the section thickness sampling fraction was 1. In all animals, 30 µm sections, each 180 µm apart, were analysed; thus, the fraction of sections sampled was 30 µm /180 µm = 0.167. The number of neurons in the analysed region was estimated by multiplying the number of neurons counted within the sample regions by the reciprocals of the area sampling fraction and the fraction of the section sampled.

### Immunofluorescence on brain sections

Animals were perfused 4 or 28 days after completing MPTP treatment, brains were processed as described above, and coronal brain sections were obtained (30 μm thickness). Sections were permeabilised with 1% Triton X-100 in PBS for 1 h and blocked in 5% (w/v) BSA, and 1% Triton X-100 in PBS for 1 h. Specimens were then incubated overnight at 4 °C with the following primary antibodies in different combinations: sheep-derived anti-TH (1:1000, Novus Biologicals-Biotechne, Minneapolis, MN, USA), rabbit-derived anti-cleaved-caspase-3 (cl-Casp3, 1:250, Cell Signaling Technologies, Danvers, MA, USA), rabbit-derived anti-cleaved-poly (ADP-ribose) polymerase (cl-PARP, 1:800, Cell Signaling Technologies), rabbit-derived anti-Ionized calcium-binding adapter molecule 1 (IBA-1, 1:500, Fujifilm Wako, Osaka, Japan), rabbit-derived anti-phosphorylated mixed lineage-kinase domain like (pMLKL, 1:500, Abcam, Cambridge, UK), mouse-derived anti-CD68 (1:250, Invitrogen-Thermo Fisher Scientific, Waltham, MA, USA), sheep-derived anti-TREM2 (1:250, R&D Systems-Biotechne, Minneapolis, MN, USA), goat-derived anti-GAL3 (1:500, R&D Systems) and rat-derived anti-GAL3 (1:250, Millipore-Merck, Darmstadt, Germany). Antibodies were diluted in 1% BSA and 1% Triton X-100 in PBS. Sections were then rinsed for 1 h in PBS containing 0.1% Triton X-100, incubated for 1 h with the secondary antibodies Alexa Fluor^®^ 488, Alexa Fluor^®^ 546, and Alexa Fluor^®^ 647 (1:500, Invitrogen), that were diluted in 1% BSA, 0.1% Triton X-100 in PBS, washed again with 0.1% Triton X-100 in PBS for 1 h, and finally mounted in 50% glycerol.

For densitometric analysis of striatal TH immunoreactivity, images were acquired using an inverted ZEISS Apotome microscope (Zeiss, Oberkochen, Germany). Three images from TH-immunostained sections were taken per animal, and measurements were performed using the Fiji/ImageJ software (Rasband, W.S., ImageJ, U. S. National Institutes of Health, Bethesda, Maryland, US). The density of TH immunoreactivity in the cortex was used to subtract the general background.

For cell counting of brain sections, the images were acquired using an inverted ZEISS LSM 7 DUO confocal laser scanning microscope with exactly the same laser intensity and gain conditions. The images were quantified using ImageJ/Fiji software without any other treatment. The proportion of each morphological phenotype of microglia in the SNpc in different experimental groups and the number of GAL3^+^ and CD68^+^ cells was estimated using a cell counter plugin included in Fiji/ImageJ software.

To detect necrotic cell death, propidium iodide (PI, Thermo Fisher Scientific) labelling was used as previously described [34, 35]. Briefly, PI (10 mg/ml) was diluted in 0.9% NaCl. 20 mg/kg of PI in a total volume of not more than 100 µl was administered to mice ip 1 h before sacrifice. PI labelling was quantified automatically per ROI area.

For surface Imaris reconstruction, Z-stacks at 0.64 μm steps (1024 × 1024 resolution) taken using ZEISS LSM 7 DUO confocal laser scanning microscope at 63X, were opened in Imaris (v.9.6, Oxford Instruments) in their original format. Z-stacks were automatically reconstructed into a 3D model without further image pre-processing.

### Dopaminergic neuron primary cultures, microglial primary cultures and treatments

The primary neuronal cultures from rat ventral mesencephalon were prepared as previously described [7] with modifications. Rat embryos were recovered at day 15.5 from gestating Wistar rats, and the ventral mesencephalon was dissected in Hank’s Balanced Salt Solution (HBSS, Biowest, Nuaillé, France). Pooled tissue was rinsed three times and incubated in 0.1% trypsin/0.05% DNase/HBSS (trypsin from Biowest and DNase from Sigma Aldrich) at 37 °C for 20 min, rinsed four times in 0.05% DNase/HBSS and mechanically dissociated using a 1-ml Gilson pipette. The tissue was centrifugated at 100 g for 5 min, and the pellet was resuspended in Dulbecco’s Modified Eagle Medium (DMEM, Biowest) supplemented with 10% fetal bovine serum (FBS, Biowest), N2 (Gibco-Thermo Fisher Scientific), B27 (Gibco-Thermo Fisher Scientific) and 1% penicillin–streptomycin (Invitrogen). Cell number and viability were assessed in a hemacytometer by trypan blue dye exclusion (> 98% viable cells). A total of 200,000 cells/cm^2^ were plated on glass coverslips previously coated overnight at 4 °C with poly-L-lysine hydrobromide (100 µg/mL, Sigma Aldrich) and overnight at 37 °C with bovine fibronectin (5 µg/mL, Sigma Aldrich). Cells were grown at 37 °C, 5% CO_2,_ in a humidified incubator. After 2 days in vitro (DIV) the medium was changed to serum-free condition, and after 5 DIV half of the culture medium was replaced by fresh medium. The treatments were applied at 8 DIV for 3 days (see [7]). Cells were treated in different combinations with the neurotoxic MPTP metabolite MPP^+^ (3 µM, Sigma Aldrich), the broad-spectrum caspase inhibitor N-benzyloxycarbonyl-Val-Ala-Asp-fluoromethyl ketone (zVAD-fmk, 100 µM; R&D Systems), the caspase-8 inhibitor z-Ile-Glu(OMe)-Thr-Asp(OMe)-fluoromethyl ketone (zIETD-fmk, 100 µM, Sigma Aldrich), the caspases inhibitors vehicle dimethyl sulfoxide (DMSO, Sigma Aldrich) and the receptor-interacting protein kinase 1 (RIPK1) inhibitor necrostatin-1 (30 µM, Sigma Aldrich).

Primary microglial cells were prepared from mouse cortices, as previously described [36], and cultured for 14 days in a T75 flask before treatment. After 14 days, microglial cells were isolated, and 50,000 cells were plated in 24-well plates. Cell number and viability were assessed in a hemacytometer by trypan blue dye exclusion. The cells were grown in DMEM, 10% FBS, and 1% penicillin–streptomycin at 37 °C, 5% CO_2_ in a humidified incubator. For necroptosis induction, cells were treated with 1 µg/ml lipopolysaccharide (LPS) and 100 µM of zIETD-fmk for 48 h.

### Immunocytochemistry on cultured cells

Cell cultures of ventral mesencephalon were analysed by immunofluorescence technique for TH^+^ cells survival, nuclei morphology, and neurite length. For these analyses, experiments were repeated at least four times with cultures isolated from independent dissections. Briefly, cells were fixed with 4% PFA for 20 min, washed with PBS, and processed for immunodetection following the same procedure as for brain sections. Additionally, cells were counterstained with Hoechst 33258 (Invitrogen) to visualise cell nuclei and studied under an epifluorescence inverted ZEISS Apotome microscope.

To determine cell survival, manual counts from each condition were performed for TH^+^ neurons with visible neurites, and a total of 300-400 TH^+^ neurons were counted in the total area of the coverslip. To distinguish between apoptotic versus necrotic cell death, we observed the nuclei morphology among the TH^+^ neurons presenting shrunken cell bodies and total loss of neuritic extensions. Neurons with fragmented chromatin included in apoptotic bodies [8, 37] were considered apoptotic cells, whereas for necrosis, those with condensed or pyknotic chromatin [37, 38]. The total number of cells per well with either apoptotic or necrotic cell morphology was counted. As an early sign of neurodegeneration, neurite length analysis was performed for TH-stained neurons. We randomly selected a minimum of 30 TH^+^ neurons among the cells with intact nucleus morphology (non-fragmented or condensed) and analysed them with the Simple Neurite Tracer Fiji/ImageJ plugin to determine the average length of neurites per cell. For TH^+^ cells survival and neurite length analysis, results were expressed as the percentage of each condition compared to the control situation, whereas for fragmented or condensed nuclei, absolute values were presented.

Necroptosis induction in primary microglia was analysed by immunofluorescence technique against pMLKL and IBA-1. Additionally, cells were treated with 1 µg/ml PI for 10 min previously to fixation to detect cell membrane permeabilisation.

### Cell lines culture

Murine microglial BV2 cell lines were cultured in DMEM-GlutaMax (Gibco) supplemented with 10% FBS and 1% penicillin-streptomycin and kept at 37 °C 5% CO_2_. Galectin-3 knockout BV2 (BV2Gal3KO) cells were generated with the Alt-R CRISPR-Cas9 System (Integrated DNA Technologies, IDT), following manufacturer’s instructions. Briefly, two Alt-R CRISPR-Cas9 CRISPR RNA (crRNA, IDT) guides were designed to excise the transcription starting site of the *Lgals3* (galectin-3) gene (guide 1: CTTCTAGTACTCTTTTCCCC, guide 2: TACCCGCAGGAGAGCCAAGG; protospacer-adjacent motif sequences not included). Each one was then mixed with trans-activating Alt-R® CRISPR-Cas9 CRISPR RNA (tracRNA, IDT) to form the single-guide RNA (sgRNA). Afterward, ribonucleoprotein (RNP) complex was formed by mixing Alt-R S.p. HiFi Cas9 Nuclease V3 (IDT) with both sgRNAs in OptiMEM (Gibco). Reverse transfection was performed by mixing the RNP complex with Lipofectamine RNAiMAX (Invitrogen) and added to a 96-well plate, where 40.000 BV2 cells were then seeded. The same procedure was carried out as negative control with the RNP complex without the crRNA (WT cells). After that, PI (Biolegend 421301)-negative single-cells (1:1000) were sorted using the BD FACSAria™ III Cell Sorter (BD Biosciences). *Lgals3* ablation was further validated by RT-PCR analysis (data not shown).

Rat dopaminergic N27 cells (RRID:CVCL_D584) were used for in vitro assays. Cells were maintained in RPMI 1640 media (Gibco, Thermo Fisher Scientific) supplemented with 1% penicillin–streptomycin (Sigma-Aldrich) and 10% FBS (Gibco, Thermo Fisher Scientific).

### Quantification of phagocytosis in vitro

N27 cells were seeded on 12-well plate at 100,000 cells/well in RPMI 1640 media with 1% penicillin–streptomycin and 10% FBS. Later, cells were challenged with MPP^+^ at 200 µM or MPP^+^ and pan-caspase inhibitor zVAD-fmk at a concentration of 50 µM. After 24 h, the media was carefully collected including non-adherent cells. Adherent cells were collected after trypsin incubation for 5 min and mixed in the previous tube. So as, all cells in the well were collected, regardless of their adherent ability. N27 cells were then centrifuged and resuspended in DMEM 1% penicillin–streptomycin. Total cells were counted and stained with CFSE dye following the manufacturer recommendations (Cayman Chemicals, Ann Arbor, MI, USA).

On the other hand, BV2WT and BV2Gal3KO cells were seeded on 96-well PhenoPlate (Revvity, Waltham, MA, USA) in DMEM 1% penicillin–streptomycin supplemented with 10% FBS at a density of 4000 cells per well. 24 h after seeding, cells were stained with MitoTracker Deep Red at 1:2000 dilution for 20 min (Thermo Fisher Scientific) followed by Hoechst 33258 at 1:1000 dilution for 10 min (Thermo Fisher Scientific). After 2 washes in DMEM 1% penicillin–streptomycin, 4000 pretreated and prestained N27 cells were added per well. Additionally, BV2Gal3KO cells were supplemented with recombinant GAL3 at a concentration of 300 nM.

Recombinant human GAL3 was produced by the Lund Protein Production Platform (LP3) as previously described [39] and diluted in sterile PBS.

Cells were either fixed after 3 h of co-culture or tracked for 6 h at 37 °C, 5% CO_2_ in Operetta CLS High-Content Analysis System (Revvity) under a 40X objective.

For phagocytosis analysis, we took advantage of Harmony Software (Revvity). Briefly, nuclei were first identified through Hoechst staining. Then, nuclear CFSE staining was used to discriminate between N27 neurons (positive) and BV2 microglial cells (negative). MitoTracker was used to determine the cytoplasm. BV2 cells with at least one spot of CFSE staining exclusively in the cytoplasm were defined as phagocytic BV2 cells (see Fig. 8c). Cells were fixed for 10 min with 4% paraformaldehyde at RT. The percentage of phagocytic BV2 cells per total BV2 cells was quantified. Additionally, Annexin-V-APC (BioLegend, San Diego, CA, USA) was used to determine the phosphatidylserine exposure, Annexin-V was diluted 1:25 in Annexin-V Binding Buffer (BD Biosciences, Franklin Lakes, NJ, USA) and incubate for 20 minutes. Later, Annexin-V was further diluted to 1:100 during imaging. For live-cell tracking, the area under the curve was calculated and plotted using GraphPad Prism®. At least three replicates per group were analysed for live-cell experiments and a minimum of seven replicates per group were analysed for fixed experiments.

### Measurement of striatal dopamine

The analysis of striatal dopamine (DA) was performed by means of HPLC equipped with a VWR-Hitachi Elite Lachrom L-2130 pump in conjunction with a glassy carbon electrode set at -550 mV (DECADE II, ANTEC, Leyden, Netherland) 28 days after MPTP treatment. A Merck LiChroCART cartridge (125 mm × 4 mm) column filled with LiChrospher reversed-phase C18 5 µm material was used. The mobile phase consisted of a mixture of 0.05 M of sodium acetate, 0.4 mM of 1-octanesulfonic acid, 0.3 mM of Na_2_EDTA, and 70 ml methanol, adjusted to pH 4.1 with acetic acid. All reagents and water were HPLC grade. The flow rate was 1.0 ml/min. All molecules in fresh tissue were measured according to the method previously described [40]. The concentration of striatal DA was calculated with the aid of eDAQPowerChrom 280 software.

### Real-Time RT-PCR

Animals used for RT-PCR were sacrificed by decapitation the fourth day after last MPTP injection. SN was dissected from each mouse, snap frozen in liquid nitrogen, and stored at -80 °C. Total RNA was extracted from the mouse SN of different groups using the RNeasy^®^ kit (Qiagen, Venlo, Netherlands). cDNA was synthesised from 1 μg of total RNA using Revert Aid First Strand cDNA Synthesis Kit (Thermo Fisher Scientific) in 20 μl reaction volume as described by the manufacturer. At least four animals per group were used.

Real-time PCR was performed using 5 μl SensiFAST™ SYBR NO-ROX KIT (Bioline, London, UK), 0.4 μl of each primer at a concentration of 10 μM, and 4.2 μl cDNA to get to a final reaction volume of 10 μl for a 384-well plate. Negative controls without cDNA were included. Amplification was run in a Lightcycler® 480 Instrument II (Roche, Basel, Switzerland) thermal cycler at 95 °C for 2 min, followed by 40 cycles consisting of a denaturation phase for 5 s at 95 °C, followed by a second phase of hybridisation at 65 °C for 10 s, and a final phase of elongation at 72 °C for 20 s. The process was terminated by a final step of 5 min at 72 °C. Melting curve analysis confirmed a single PCR product. β-actin gene (*Actb*) served as a reference gene and was used for sample normalisation. The cycle at which each sample crossed a fluorescence threshold (Ct value) was determined, and the triplicate values for each cDNA were averaged. The primer sequences are: *Actb* (F:5´- GGCTATGCTCTCCCTCACG and R: 5´-CTTCTCTTTGATGTCACGCACG), *Lgals3* (F:5´-GATCACAATCATGGGCACAG and R: 5´- GTGGAAGGCAACATCATTCC), *Trem2* (F:5´- GTTTCTTGCAGCCAGCATCC and R: 5´-GGGTCCAGTGAGGATCTGAAG), *Cd68* (F:5´- GCCCAAGGAACAGAGGAAG and R: 5´- GGCTGTAGGTGTCATCGTG), *Clec7a* (F:5´-CTGGTATGGAAGTAAGAGACACTGC and R: 5´- CGGTGAGACGATGTTTGGC), and *Cx3cr1* (F:5´- GCCTCCACAATGCCATGTGC and R: 5´-GCTGCACTGTCCGGTTGTTC).

### Statistical analysis

An assessment of normality of data by Shapiro-Wilk analysis was performed to allow parametric testing. Differences between groups were examined for statistical significance using one-way analysis of variance (ANOVA), followed by Fisher’s LSD *post hoc* analysis, with α = 0.05. The data were analysed with a computer software system (StatView 5.0; Abacus Concepts, Berkeley, CA). A *p*-value < 0.05 denoted a statistically significant difference.

## Results

### The specific deletion of *Casp3* in TH^+^ neurons does not protect from MPTP intoxication in the nigrostriatal pathway

Previous studies have clearly shown the involvement of apoptotic cell death in the neurodegeneration of SNpc dopaminergic neurons of MPTP-treated mice in the subacute dosage regime (30 mg/kg/day for five consecutive days) [10, 31]. We aimed to study if specific gene deletion of *Casp3*, the most relevant executioner caspase in apoptosis, an immunologically silent form of cell death, is able to prevent the death of the SNpc dopaminergic neurons in this MPTP model or alternatively promotes a switch towards an immunogenic form of cell death. With that goal, our group has recently established a conditional mouse model deleting *Casp3* in TH^+^ neurons (TH-C3KO) that allows us to study the role of caspase-3 specifically in the cell death process of those neurons after MPTP treatment (Fig. 1a) [29]. The experimental design applied for both WT and TH-C3KO genotypes is shown in Fig. 1b. Firstly, we studied the potential occurrence of apoptosis in the ventral mesencephalon from MPTP-intoxicated mice. Activation and cleavage of caspase-3 in the subacute MPTP model has been found at early time points ranging from hours after the second dose of the toxin [13] to 4 days after the last one [11]. We first evaluated the presence of cleaved caspase-3 in WT mice dopaminergic neurons by immunofluorescence after the third, fourth and fifth MPTP injections. In accordance with a previous study [13], we found a maximum number of apoptotic dopaminergic cells within the SNpc of WT mice hours after the third day of MPTP administration (arrows in Fig. 1c), with no evidence of cleaved caspase-3 staining in saline-injected mice. Thus, we next analysed the presence of cleaved caspase-3 on the third day after MPTP administration in TH-C3KO/MPTP mice. Notably, TH-C3KO/MPTP mice failed to present cleaved caspase-3 staining in TH^+^ neurons, supporting the effective deletion of *Casp3* gene in our mouse model and indicating a disruption in the apoptotic cascade. However, apoptosis could be executed through other caspases beyond caspase-3, converging in the cleavage of PARP [41, 42]. Despite PARP could be cleaved by a broad subtypes of proteases, the generation of 89 kDa C-terminal fragment is specific of caspase activation [43]. We used a specific antibody recognizing the presence of such PARP fragment after the third day of MPTP administration in the different experimental groups. Similarly to what was observed for caspase-3, cleaved-PARP was found in dopaminergic neurons within the SNpc of WT/MPTP mice (Suppl. Figures 1a and 1b). Contrarily, we found no cleaved-PARP staining in TH-C3KO/MPTP TH^+^ neurons, thus supporting the view that caspase-3 acts as the main effector killing caspase of apoptosis in the subacute MPTP regime.

**Fig. 2:**
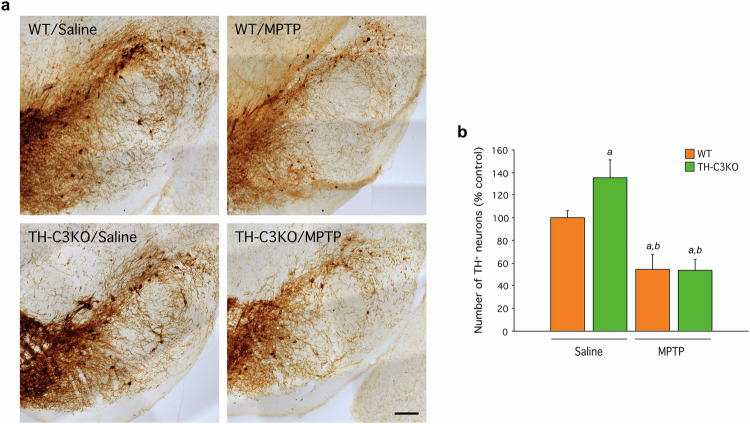
Effect of MPTP on the survival of SNpc dopaminergic neurons of WT and TH-C3KO animals. **a** Coronal sections of SNpc immunostained for TH of WT and TH-C3KO animals injected with saline solution or MPTP and analyzed 28 days after the last injection. Decreased TH^+^ cell number is evident in both WT/MPTP and TH-C3KO/MPTP. Scale bar, 200 µm. **b** Stereological analysis of the survival of SNpc dopaminergic neurons. Absolute values of TH^+^ cells were as follows: WT/saline: 4942 ± 311; TH-C3KO/saline: 6705 ± 754; WT/MPTP: 2704 ± 654; TH-C3KO/MPTP: 2636 ± 463. Data represent the mean ± SEM from at least four animals. Statistical analysis: One-way ANOVA followed by the Fisher’s LSD *post hoc* test for multiple comparisons was used, with α = 0.05: (**a**), compared with the WT/saline group; (**b**), compared with the TH-C3KO/saline group; *p* < 0.05.

To examine the long-term effect of apoptosis ablation, a stereological analysis was performed 28 days after the MPTP treatment period on TH-immunostained SNpc sections from WT and TH-C3KO mice (Fig. 2a, b). We observed that the MPTP treatment caused a 45% significant reduction of TH^+^ neurons in WT mice compared to the saline group. Importantly, *Casp3* deletion failed to confer protection against MPTP-induced neurotoxicity as a similar number of remaining TH^+^ neurons was found in TH-C3KO/MPTP compared to WT/MPTP group. In the TH-C3KO/saline group, an increase in TH^+^ neurons was also found compared to the WT/saline group, as we have previously described [29].

Furthermore, we measured the striatal fiber density of TH-stained sections by immunofluorescence in WT and TH-C3KO animals injected with saline or MPTP (Suppl. Figures 2a and 2b). At 28 days after the MPTP treatment, for both WT and TH-C3KO groups, significant decreases in TH fiber density compared to their respective saline groups were observed. This further supported the view that the absence of caspase-3 is not neuroprotective against the MPTP-induced death of nigro-striatal dopaminergic neurons. We extended this analysis to the shorter time point after the MPTP treatment (4 days), and we found more dramatic decreases in TH^+^ fiber density in WT and TH-C3KO mice treated with MPTP than in the 28-days time point. This suggests that, at longer time points after MPTP administration, sprouting of dopaminergic striatal terminals is occurring, a phenomenon previously observed by other authors [44]. In the TH-C3KO/saline group, a significant decrease in striatal fiber density was also found compared to the WT group, as we have already described [29].

In this line, analysis of DA levels by HPLC of striatum 28 days after MPTP treatment corroborated fiber density results (Suppl. Figure 2c). MPTP caused a dramatic decrease of DA levels compared to control values in both genotypes. Strikingly, TH-C3KO/saline animals presented a mild increase in the DA striatal content as compared to WT/saline that could underline a decreased DA release or an increased compensatory synthesis. Overall, these results reveal that the absence of caspase-3 does not prevent either dopaminergic neuronal death in the cell bodies located in the SNpc or loss of dopaminergic innervation in the neuronal terminals located in the striatum in response to MPTP administration and does not affect striatal sprouting-based regeneration.

### In absence of apoptosis, signs of necrosis appear in SNpc inducing microglia phagocytosis after MPTP treatment

**Fig. 3:**
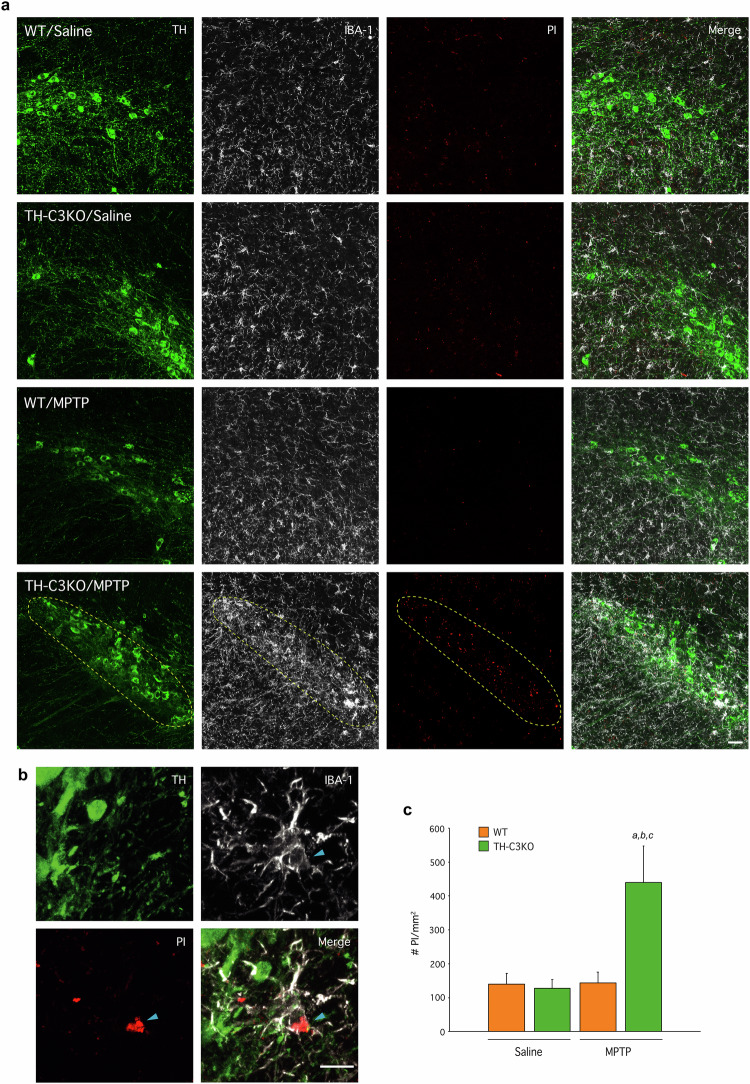
Induction of necrosis and microglia activation in dopaminergic neurons of SNpc of TH-C3KO animals after MPTP treatment. **a** Coronal sections of SNpc showing neurons immunostained for TH, IBA-1 and presenting PI fluorescence of WT and TH-C3KO animals injected with saline solution or MPTP and analysed 4 days after the last injection. Yellow dashed lines highlight presence of PI and higher expression of IBA-1 in SNpc area in TH-C3KO/MPTP compared to other conditions. Scale bar, 30 µm. **b** Higher magnification area of TH-C3KO/MPTP condition with arrow pointing IBA-1^+^ cells containing PI-stained material. Scale bar, 10 µm. **c** Quantification of PI particles per mm^2^. Data represent the mean ± SEM from at least three animals. Statistical analysis: One-way ANOVA followed by the Fisher’s LSD *post hoc* test for multiple comparisons was used, with α = 0.001. (**a**), compared with the WT/saline group; (**b**), compared with the TH-C3KO/saline group; (**c**), compared with the WT/MPTP; *p* < 0.05.

As we were unable to observe apoptotic dopaminergic neurons in TH-C3KO/MPTP after MPTP treatment and considering that *Casp3* gene deletion failed to confer long-term protection against MPTP, we reasoned that dopaminergic neurons could have switched the cell death pathway in the absence of caspase-3. Indeed, different in vitro approaches have established that failure to activate caspases may change the cell death fate from apoptosis to necrosis [45, 46]. Contrary to apoptosis, necrosis entails plasma membrane rupture and permeabilisation, which can be easily recognised in vivo based on cell internalisation and DNA binding of previously administrated PI. Thus, we performed *premortem* injections of PI and analysed its presence in the ventral mesencephalon together with immunostaining of TH and the pan-microglial marker, IBA-1 (Fig. 3a). We barely detected the presence of PI in WT/saline, TH-C3KO/saline and WT/MPTP groups. However, we did observe an evident PI-stained area in the TH-C3KO/MPTP group that was strictly confined to the SNpc. Interestingly, most of the PI staining was associated with IBA-1-labelled viable cells (arrow in Fig. 3b), which suggests microglial phagocytosis of nuclear and cytosolic debris of necrotic cells was taken place. Imaris^®^ 3D video-reconstruction confirmed the localisation of PI within IBA-1 cells (Suppl. Video 1) with no signs of microglial degeneration. We quantified the number of PI particles per SNpc area in the four animal groups and found a significant increase in the TH-C3KO/MPTP group compared to others (Fig. 3c), suggesting that apoptosis in those mice was replaced by necrosis as the prevailing cell death mode of dopaminergic neurons. Furthermore, microgliosis of IBA-1-stained cells was evident in the SNpc of TH-C3KO/MPTP animals, which would suggest microglia activation was promoted. This feature, however, was not observed in WT/MPTP mice (Fig. 3a), which is in accordance with the immunogenic nature of necrotic cell death observed in TH-C3KO/MPTP mice. Although we were unable to find any colocalisation of PI with the neuronal marker TH, the decreased number of TH neurons in the SNpc, the increased microgliosis, and the presence of PI inside microglial cells strongly point to a neuronal necrotic process.

**Fig. 4:**
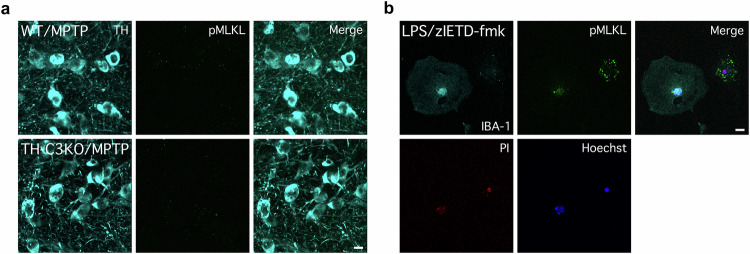
Absence of pMLKL immunostaining after MPTP treatment in SNpc of WT and TH-C3KO animals. **a** Coronal sections of SNpc immunostained for TH and pMLKL of WT and TH-C3KO animals injected with MPTP and analysed 4 days after the last injection. No positive cells for pMLKL were observed. Scale bar, 10 µm. **b** As positive control of pMLKL inmmunostaining primary microglia cultures were treated for necroptosis induction with LPS-zIETD-fmk and immunostained with pMLKL, IBA-1, PI and Hoechst 33258. Scale bar, 20 µm.

Necroptosis is another form of regulated cell death with the morphological features of necrosis, including PI staining, but relying on an orderly programmed molecular cascade. Phosphorylation of MLKL (pMLKL) is the final event responsible for cell membrane permeabilisation, being considered a hallmark of necroptosis. To further characterise the cell death mechanism of dopaminergic neurons in our model, we performed immunostaining of pMLKL and TH in the ventral mesencephalon from both WT-MPTP and TH-C3KO/MPTP mice, but we were unable to find a single dopaminergic neuron exhibiting pMLKL staining (Fig. 4a), nor any sign of increased pMLKL immunoreactivity in the SNpc area. Notably, we validated our pMLKL staining protocol using an experimental condition which was previously described to trigger programmed necrosis such as a primary culture of microglia cells treated with LPS and zIETD-fmk (caspase-8 inhibitor) [47] (Fig. 4b). In this case, we did observe IBA-1^+^ cells showing pMLKL staining, PI incorporation, and nuclear condensation, thus validating the reliability of our pMLKL staining protocol. These results indicate that the death of dopaminergic neurons in our model relies on a different signalling cascade from necroptosis.

### Primary cultures of dopaminergic neurons undergo necrotic but not necroptotic cell death under MPP^+^ and caspases inhibitors treatment

**Fig. 5:**
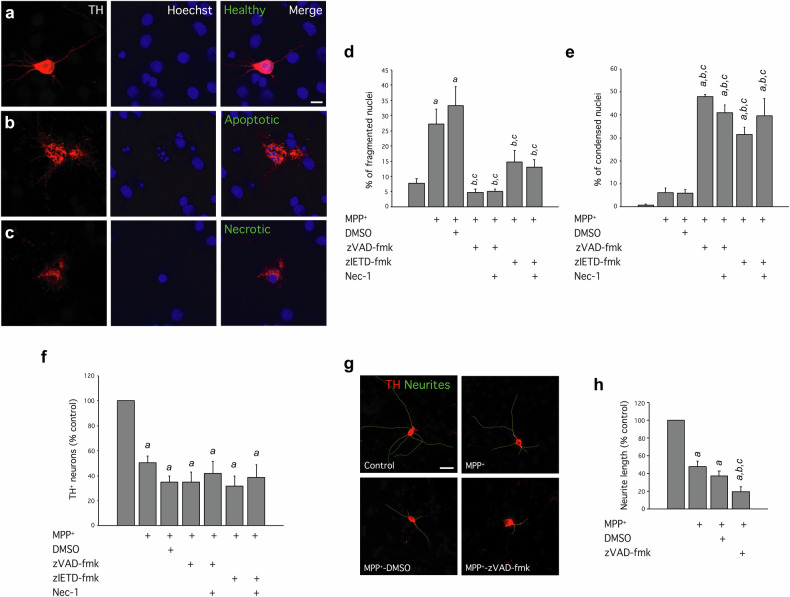
Effect of MPP^+^, caspase inhibition and RIPK1 inhibition on different aspects of primary cultures of mesencephalic dopaminergic neurons such as nuclei morphology, cell survival and neurite length. Cultures were treated with 3 μM MPP^+^; 3 μM MPP^+^, and DMSO, as caspase inhibitors vehicle; 3 μM MPP^+^, and 100 μM zVAD-fmk; 3 μM MPP^+^, and 100 μM zIETD-fmk; 3 μM MPP^+^, 100 μM zVAD-fmk, and 30 µM Nec-1; or 3 μM MPP^+^, 100 μM zIETD-fmk and 30 µM Nec-1. **a**–**c** Morphological assessment of nuclei in dopaminergic neurons. Representative examples of dopaminergic neurons with intact nuclei (**a**), and fragmented (**b**) or condensed nuclei (**c**) as representative of apoptotic and necrotic cells respectively, are shown. Scale bar, 10 µm. **d** Mean percentage of dopaminergic neurons with fragmented (apoptotic) nuclei. **e** Mean percentage of dopaminergic neurons with condensed (necrotic) nuclei. **f** Mean percentage of surviving dopaminergic neurons. Values from each treatment were expressed as a percentage of the untreated control (100%). **g** Neurite length assessment in TH^+^ neurons. Representative examples of the different conditions are shown with outlined neurites in green. Scale bar, 30 µm. **h** Values of neurite length from each condition were expressed as a percentage of the untreated control (100%). Data represent the mean ± SEM from at least four independent experiments. Statistical analysis: One-way ANOVA followed by the Fisher’s LSD *post hoc* test for multiple comparisons was used, with α = 0.05; (**a**), compared with the control group; (**b**), compared with the MPP^+^ group; (**c**), compared with the MPP^+^/DMSO group; in panels (**d**, **e**, **h**, *p* < 0.05; in panel **f**, *p* < 0.001.

The signs of necrotic death previously found in the SNpc of our mice and the lack of pMLKL labelling suggest a necrotic death of dopaminergic neurons. However, some authors have previously reported necroptotic cell death induction in animal models of PD based on MPTP injections [48, 49], while others failed to observe it [50]. To further clarify the occurrence of necroptosis, apoptosis or necrosis in dopaminergic neurons undergoing complex I inhibition, we used an additional in vitro model. As caspase inhibition is a key driver of necroptosis in many cellular models, we used primary mesencephalic cultures that were subjected to MPP^+^ treatment alone or in combination with pan-caspase/caspase-8 inhibition, a condition that has been shown to cause a switch in of the cell death mode of dopaminergic neurons from apoptosis to necrosis [7]. Thus, primary dopaminergic neurons were exposed for 72 h to 3 µM MPP^+^, as this concentration has been described to cause the death of dopaminergic neurons by apoptosis [8, 14, 51]. We used Hoechst 33258 staining as a helpful tool for studying the cell death process distinguishing apoptosis vs necrosis [52]. Fragmented nuclei are easily observed at the later stage of apoptosis [8, 37], whereas, during necrosis, condensed nuclei are usually observed [7, 37, 38]. In our in vitro model, healthy TH^+^ neurons presented intact nuclei morphology and long neuritic extensions (Figs. 5a, g). In contrast, for neurons in the dying process, neurite arborisation loss was evident, besides presenting either a fragmented (Fig. 5b), or a condensed nucleus (Fig. 5c), indicative of apoptosis or necrosis, respectively. Most of the TH^+^ neurons in the control condition possessed intact nucleus morphology with a low number of neurons in which we could observe neurite retraction and fragmented (Fig. 5d) or condensed nuclei (Fig. 5e). MPP^+^ or MPP^+^/DMSO treatment caused a decrease of more than 50% in the number of viable dopaminergic neurons (Fig. 5f) accompanied by a relevant increase of neurons displaying fragmented nuclei (Fig. 5d), and no significant change in condensed nuclei proportion (Fig. 5e), pointing out apoptosis as the main mechanism responsible for neuronal death. In line with our in vivo genetic model, the simultaneous treatment of MPP^+^ and zVAD-fmk or zIETD-fmk, pan-caspase and caspase-8 inhibitors, respectively, failed to prevent the MPP^+^-induced cell death (Fig. 5f) triggering a switch in the cell death pathway towards a predominant necrosis, as an increase in the number of condensed nuclei was the only observed change in nuclei morphology compared to control cells (Figs. 5d, e). This was particularly more evident after zVAD-fmk treatment, which triggers a pan-caspase inhibition that includes caspase-3 inhibition.

Next, we wanted to confirm if this redirection of the cell death pathway of dopaminergic neurons involves regular necrosis rather than induction of the necroptotic program, as observed in the in vivo model. In cells undergoing necroptosis, RIPK1 activation is critical for necrosome assembly and cell death execution [53]. Thus, we took advantage of necrostatin-1 (Nec-1), a RIPK1 inhibitor, to confidently assess the occurrence of necroptosis in response to MPP^+^ in combination with either zVAD-fmk or IETD-fmk. Treatment with Nec-1 failed to confer protection for cell survival of dopaminergic neurons in MPP^+^/zVAD-fmk and MPP^+^/zIETD-fmk conditions (Fig. 5f) as well as any significant effect in nuclei morphology (Fig. 5d, e).

As pointed before, neuritic retraction can be observed in response to cell death conditions. Therefore, we evaluated neurite length as an early sign of neurotoxicity preceding cell death (Fig. 5g, h). We compared neurite length in control, MPP^+^, MPP^+^/DMSO, and MPP^+^/zVAD-fmk treated cells (Fig. 5h). MPP^+^ and MPP^+^/DMSO treatment caused a significant reduction in the average neurite length compared to the control condition, while pan-caspase inhibition further reduced neurite length. This observation suggests that caspase inhibition worsens neuronal functionality in response to MPP^+^.

Altogether, these in vitro results indicate that caspase inhibition does not confer neuroprotection against MPP^+^-induced apoptotic death of mesencephalic dopaminergic neurons. Instead, dopaminergic neurons switched the cell death program from apoptosis to necrosis but not necroptosis, as Nec-1 failed to increase neuronal viability. Indeed, we provide evidence supporting that dopaminergic neurons are resistant to undergoing necroptotic cell death, even after concomitant caspase-8 inhibition (a prototypical necroptotic stimulus) and MPP^+^ treatment.

### Necrotic dopaminergic neurons induce microglia activation in vivo

**Fig. 6:**
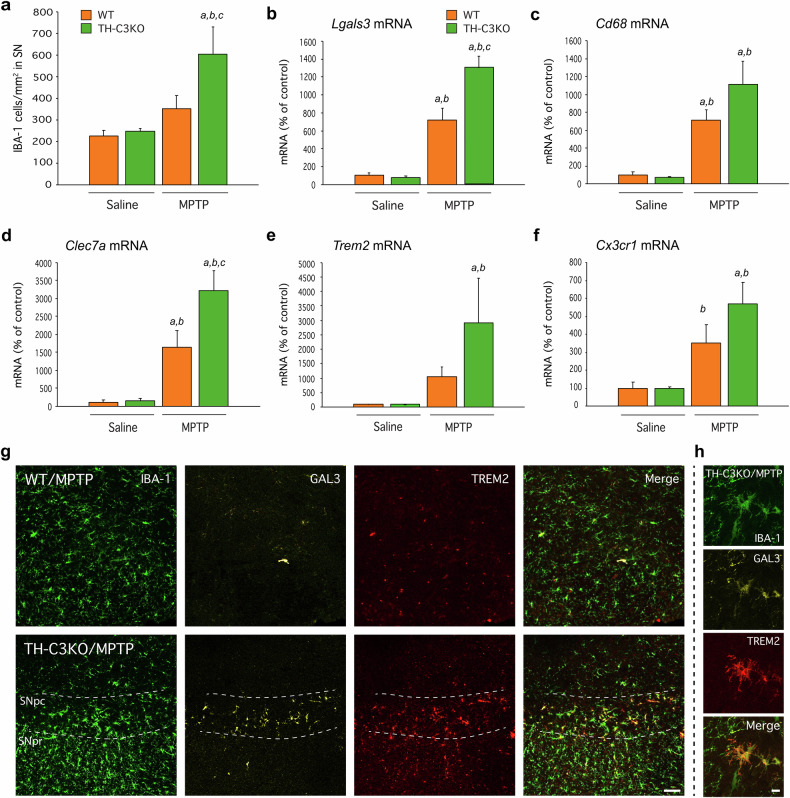
Effect of MPTP on microglia activation status, mRNA expression levels and protein expression of microglia MGnD makers of SNpc of WT and TH-C3KO animals. **a** Quantification of IBA-1^+^ cells/mm^2^ in immunostained sections of WT and TH-C3KO animals injected with saline solution or MPTP and analysed 4 after the last injection. **b**–**f** mRNA expression levels of *Lgals3*, *Cd68*, *Clec7a*, *Trem2*, and *Cx3cr1* in SNpc of WT and TH-C3KO animals injected with saline solution or MPTP and analyzed 4 days after the last injection. **g** Coronal sections of SNpc showing immunostained microglia cells for IBA-1, GAL-3 and TREM2 of WT and TH-C3KO animals injected with MPTP. Scale bar, 100 µm. Dashed lines delimit areas of SN *pars compacta* and SN *pars reticulata.* **h** Higher magnification area of TH-C3KO/MPTP condition. Scale bar, 10 µm. Data represent the mean ± SEM from at least four independent experiments. Statistical analysis: One-way ANOVA followed by the Fisher’s LSD post hoc test for multiple comparisons was used, with α = 0.05. (**a**), compared with the WT/saline group; (**b**), compared with the TH-C3KO/saline group; (**c**) compared with the WT/MPTP; in (**a**–**c**, *p* < 0.01; in **d**–**f**, *p* < 0.05.

Necrotic cell death is a proinflammatory status in nature due to the release of intracellular content. Then, we were prompted to measure the presence and activation state of microglia in SNpc after MPTP administration in WT and TH-C3KO animals. The density of IBA-1^+^ cells was quantified in SNpc (Figs. 3a and 6a). We observed a significant increase of microglial cells in the SNpc of TH-C3KO mice at 4 days of MPTP post-injection (Fig. 6a). In contrast, number of microglial cells in WT/MPTP was similar to the saline-injected groups, in line with previous observations made in the subacute MPTP model (Fig. 6a) [54]. These results agree with the non-immunogenic nature of apoptotic cell death. Next, we explored the different morphological phenotypes of microglia in the SNpc within the experimental groups. We defined three microglia subtypes: i) homeostatic microglia presenting thin cytoplasmic ramifications, and low to moderate IBA-1 expression, ii) hypertrophic microglia, characterised by hyper-ramified morphology and thickening of cytoplasmic processes, as well as increased body size and enhanced IBA-1 expression, and iii) microglia on final activation stage showing complete retraction of cytoplasmic processes and amoeboid morphology with high levels of IBA-1 expression. While no change in microglia morphology was observed in the WT/MPTP group, in the TH-C3KO/MPTP condition, we observed a significant decrease in homeostatic cell type and an increase of both hypertrophic and amoeboid microglia (Suppl. Figure 3a). Altogether, these results support the appearance of distinctive microglia subtype in TH-C3KO/MPTP mice. Besides, we extended the same analysis on microglia to the striatal terminal area (Suppl. Fig. 3b, c). Both in WT/MPTP and TH-C3KO/MPTP groups, we did not observe signs of microgliosis (Suppl. Figure 3c). This same result has been previously described for the WT/MPTP condition [54]. Regarding microglial morphology, only a moderate decrease in homeostatic microglia in the TH-C3KO/MPTP group was found compared to the TH-C3KO/saline group (Suppl. Figure 3b). Overall, these results suggest that the necrotic death of dopaminergic neurons induces the appearance of a microglia subtype distinct from homeostatic microglia, primarily observed in the SNpc based on both numerical and morphological criteria.

### TH-C3KO/MPTP condition triggers the appearance of microglial MGnD phenotype along with GAL3-dependent phagocytosis

Different microglia subtypes have been recently described in neurodegenerative conditions based on their gene expression [55]. To further investigate microglia activation, we measured the mRNA expression levels of key genes representative of the DAM/MGnD phenotype, such as *Lgals3, Cd68, Clec7a*, and *Trem2* (Fig. 6b–e) [23, 24] and *Cx3cr1* (Fig. 6f) as a marker of homeostatic microglia. Bulk mRNA analysis of the SN of mice showed that DAM/MGnD microglia markers were upregulated in the WT/MPTP condition. Expression levels of *Lgals3, Cd68*, and *Clec7a* genes were robustly upregulated in the SN of those mice compared to the saline condition (Fig. 6b–d). Importantly, in the TH-C3KO/MPTP condition, *Lgals3* and *Clec7a* were further induced when compared to WT/MPTP group (Fig. 6b, d). In the case of *Trem2*, we detected a notable increase in its transcription levels in TH-C3KO/MPTP mice, but no difference was found compared to WT/MPTP group (Fig. 6e). The same applies for *Cd68* (Fig. 6c). Interestingly, the *Cx3cr1* gene also showed upregulation in the TH-C3KO/MPTP group (Fig. 6f), which correlates with the microgliosis previously observed by immunofluorescence (Figs. 3a and 6a).

**Fig. 7:**
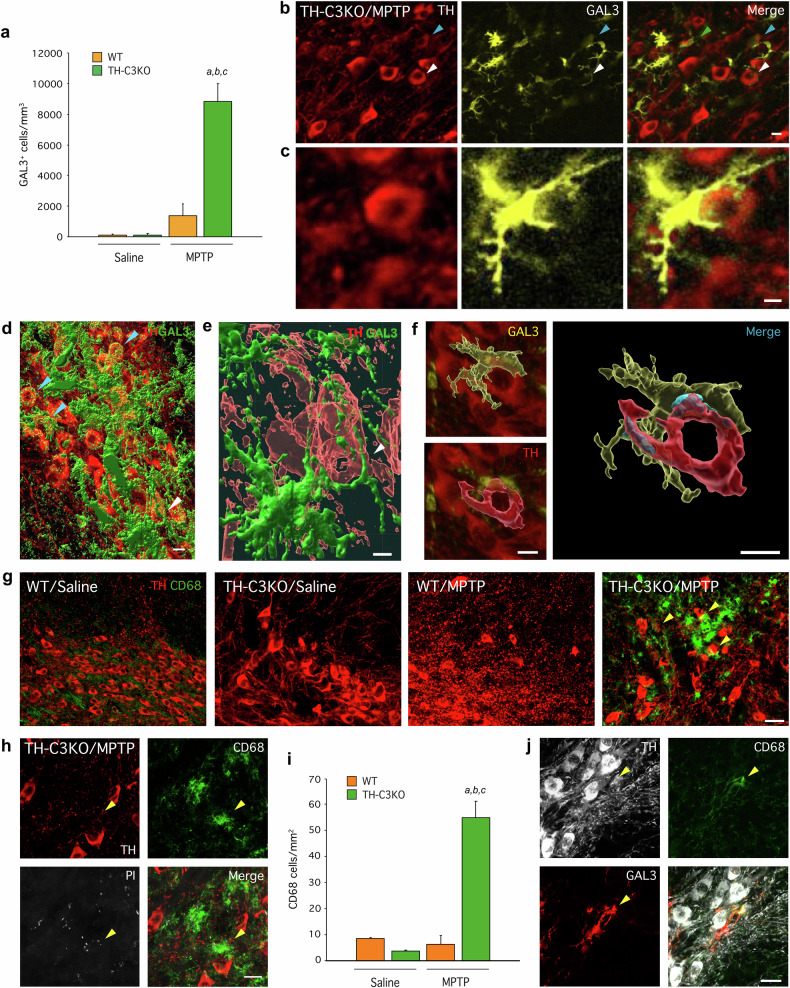
Effect of MPTP on GAL3 expressing cells and of SNpc of WT and TH-C3KO animals. **a** Quantification of GAL3^+^ cells in immunostained sections of WT and TH-C3KO animals injected with saline solution or MPTP. **b**, **c** Coronal sections of SNpc immunostained for GAL-3 and TH of TH-C3KO/MPTP condition with colored arrows pointing out different staining patterns for GAL-3 as detailed in Results section. Scale bar, 20 µm (**b**), 10 µm (**c**). **d**–**f** Imaris reconstruction analysis of coronal sections of SNpc immunostained for GAL-3 and TH of TH-C3KO/MPTP condition. In (**d**, **e**), the presence of phagocytic cups (white arrows in **d**, **e**) and dotted GAL-3 labeling on the surface of dopaminergic neurons (light blue arrows in **d**) is depicted. Scale bar, 10 µm (**d**), 5 µm (**e**). Panel (**f**) depicts a phagocytic cup revealing the presence of cell-to-cell contact in blue. Scale bar, 10 µm. **g** Coronal sections of SNpc showing immunostained cells for TH and CD68 of WT and TH-C3KO animals injected with saline solution or MPTP and analysed 4 days after the last injection. Yellow arrows mark overlapped staining areas in TH-C3KO/MPTP. Scale bar, 30 µm. **h** Higher magnification area of TH-C3KO/MPTP condition showing also PI labelling pointed by arrow. Scale bar, 20 µm. **i** Quantification of CD68^+^ cells in immunostained sections of WT and TH-C3KO animals injected with saline solution or MPTP and analysed 4 days after the last injection. **j** Coronal section of SNpc immunostained for TH, CD68 and GAL-3 of TH-C3KO/MPTP condition. Yellow arrow highlights overlapped staining area. Scale bar, 20 µm. Data represent the mean ± SEM from at least three animals. Statistical analysis: One-way ANOVA followed by the Fisher’s LSD *post hoc* test for multiple comparisons was used, with α = 0.05: (**a**), compared with the WT/saline group; (**b**), compared with the TH-C3KO/saline group; (**c**), compared with the WT/MPTP ; *p* < 0.001.

Gene expression analysis was further confirmed in WT/MPTP and TH-C3KO/MPTP animals by immunofluorescence against IBA-1, GAL3, and TREM2 (Fig. 6g). High magnification image showed colocalisation of the three markers (Fig. 6h). After MPTP treatment, microglial cells located in the SNpc from animals lacking caspase-3, but not WT, expressed high levels of GAL3 and TREM2. Quantification of GAL3^+^ microglial cells rendered a dramatic increase in TH-C3KO/MPTP animals, compared to WT/MPTP animals (Fig. 7a). Detailed analysis of TH and GAL3 immunostaining in the ventral mesencephalon of TH-C3KO/MPTP mice revealed the existence of two different conditions ascribed to GAL3^+^ microglia cells: i) cells with engulfed dopaminergic debris material (Fig. 7b, green arrow in merge panel); and ii) cells engulfing apparently healthy dopaminergic neurons recognised by the presence of phagocytic cups around such neurons (Fig. 7b, white arrow, and 7c). Strikingly, dual immunofluorescence confocal microscopy revealed the existence of diffuse GAL3 labelling on a selective population of dopaminergic neurons that were closely located or engulfed by reactive microglia (Fig. 7b blue arrow, and 7c). Imaris® 3D reconstruction confirmed observations of confocal microscopy analysis as it evidenced: i) the presence of phagocytic cups (Fig. 7d white arrow; Fig. 7e, f and Suppl. Video 2), ii) the existence of dotted GAL3 labelling on the surface of dopaminergic neurons (Fig. 7d, blue arrows) and iii) the presence of cell-to-cell (dopaminergic neuron/microglia) interactions (Fig. 7f in blue). GAL3 has been previously shown to act as an opsonin for MERTK [26], and hence, the possibility that GAL3 may be acting as opsonin for dopaminergic neurons becomes plausible. These observations highlight a prominent role of GAL3 in mediating the phagocytosis of both necrotic and apparently viable dopaminergic neurons under disease conditions.

To further investigate the role of GAL3 as an opsonin, we wondered if GAL3 upregulation, specifically in the SNpc of TH-C3KO/MPTP mice, also correlated with a higher number of phagocytic microglia. CD68 upregulation characterises microglia phagocytic phenotype [56]. We previously observed an upregulation of the *Cd68* gene in WT and TH-C3KO mice in response to MPTP (Fig. 6c). Next, we performed immunofluorescence of TH and CD68 to evaluate neuronal phagocytosis. We barely observed CD68-stained cells in WT, TH-C3KO, and WT/MPTP groups; however, in TH-C3KO/MPTP condition, we did observe CD68^+^ cells in close contact with TH^+^ neurons and some overlapped staining areas, confirming, as previously suggested, the microglial phagocytic activity over dying TH^+^ neurons or neuronal debris from degenerating neurons (yellow arrows in Fig. 7g). Supporting this observation, we detected CD68^+^ cells with dots of PI-stained material and TH^+^ neuronal debris (yellow arrows in Fig. 7h). Interestingly, we found a remarkable increase in the number of CD68^+^ cells in the SNpc of THC3KO animals but not in the WT mice after the MPTP treatments (Fig. 7i). This suggests that the phagocytic capacity of microglia in our model could be regulated by the GAL3 opsonin function. Indeed, we carried out GAL3, CD68, and TH immunostainings (Fig. 7j), and we found overlapping staining of these three markers exclusively in the TH-C3KO/MPTP group.

### GAL3 is essential for microglia phagocytic capability of necrotic dopaminergic neurons in vitro

**Fig. 8:**
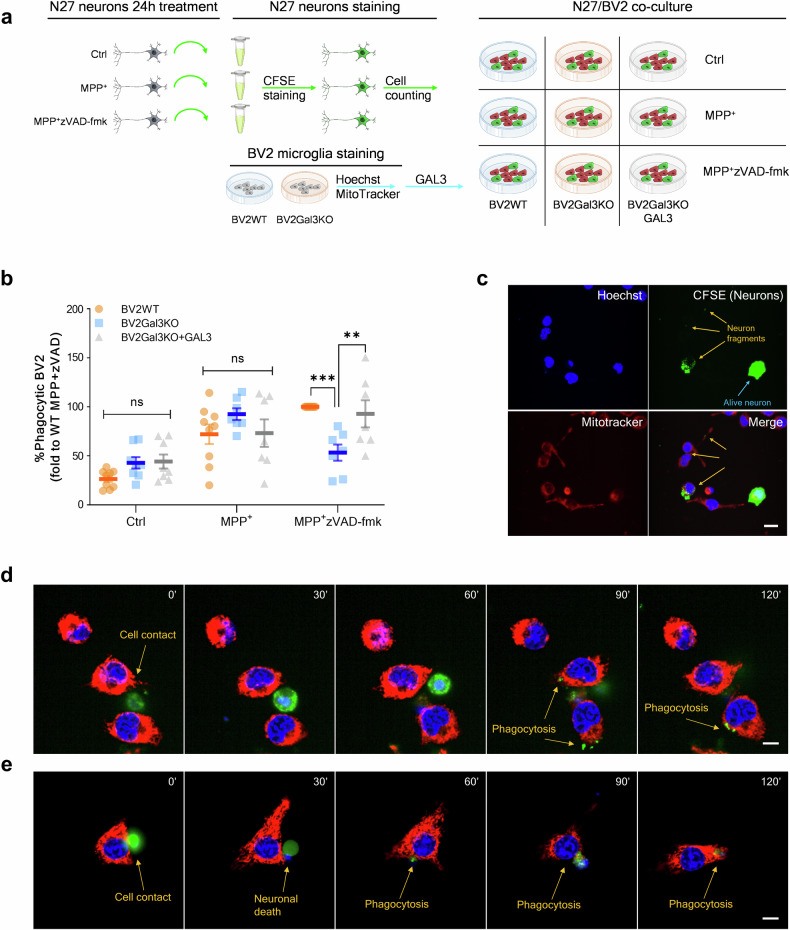
Gal3 induces the phagocytosis of stressed and dying cells after MPP^+^ and concomitant caspase inhibition. **a** Schematic representation of the in vitro phagocytosis setup. N27 dopaminergic neurons cell line was treated for 24 with MPP^+^ or MPP^+^-zVAD-fmk. zVAD-fmk was used as caspase inhibitor. N27 neurons were stained with 5-Carboxyfluorescein N-Succinimidyl ester (CFSE) before adding them to different BV2 microglia cell cultures including WT (BV2WT), Gal3KO (BV2Gal3KO), and Gal3KO supplemented with exogenous GAL3 300 nM (BV2Gal3KO + GAL3). **b** Percentage of BV2 microglial cells with at least one spot of CFSE within their cytoplasm. Data expressed as fold to WT MPP^+^-zVAD-fmk condition. **c** Representative image of counting in (**b**). Nuclei with CFSE positive staining were stablished as N27 neuronal cells. Smaller CFSE dots were identified as neuronal fragments. CFSE negative nuclei were identified as BV2 microglial cells. Mitotracker was used as cytoplasm marker. Scale bar 20 µm*.* **d**, **e** Live-cell tracking of phagocytosis was carried out in Operetta CLS High-Content Analysis System. Cell were tracked for as long as 6 h, however, shorter sequence are shown. CFSE (in green) was used to discriminate between N27 (positive) and BV2 (negative), while Mitotracker (in red) was used to define cytoplasm and Hoechst (in blue) was used to identify the nuclei. Cell contact was observed around 1 h before phagocytosis initiation. Phagocytosis of fragmented cell (**d**) and whole cell (**e**) was observed. Scale bar 10 µm. Data represent the mean ± SEM from at least seven different experiments. Statistical analysis: One-way ANOVA followed by the Fisher’s LSD *post hoc* test for multiple comparisons was used, with α = 0.05. *** represents *p* < 0.001; ** represents *p* < 0.01.

BV2 microglial cells express GAL3 even in the absence of external stimuli, thus complicating the assessment of GAL3´s potential role in promoting phagocytosis of injured or stressed neuronal cells. To address this, we generated BV2 microglial cells lacking GAL3 expression (BV2Gal3KO) and evaluated their phagocytic activity. This approach allowed us to specifically investigate the role of GAL3 as an opsonin without the confounding influence of endogenous GAL3 expression. Thus, we assessed the ability of WT BV2 (BV2WT) and BV2Gal3KO cells to phagocytose N27 dopaminergic neuronal cells in co-culture experiments, both in the presence and absence of exogenous GAL3 treatment. To replicate in vivo conditions, we challenged dopaminergic N27 neurons for 24 h with MPP^+^ or MPP^+^ plus zVAD-fmk to inhibit caspase activity. Then, neurons were collected and added to BV2WT cells, BV2Gal3KO cells, or BV2Gal3KO cells treated with exogenous recombinant GAL3 and co-cultured for 3 h (Fig. 8a). Interestingly, we first observed that Annexin-V staining of the surviving attached neurons revealed increased levels of phosphatidylserine exposure following MPP^+^, as well as after cotreatment with MPP^+^ and zVAD-fmk (Suppl. Figure 4), thus supporting a caspase-independent phosphatidylserine translocation mechanism, in agreement with previous studies [57]. Remarkably, no signs of apoptosis were detected in those neurons, indicating the presence of stressed but viable neurons and the exposure of ‘eat-me signals’ [58], a prerequisite for phagocytosis of viable cells [59]. Indeed, MPP^+^ treated N27 neurons showed an overall increase of their phagocytosis by BV2WT cells, BV2Gal3KO cells, and BV2Gal3KO cells treated with exogenous GAL3 (Fig. 8b). However, a different pattern emerged for N27 cells co-treated with MPP^+^ and zVAD-fmk (Fig. 8b). Under these conditions, BV2Gal3KO cells exhibited a significant reduction in phagocytic activity compared to BV2WT cells (Fig. 8b). Representative image shows the phagocytosis of neuronal fragments by BV2 microglia and the presence of alive N27 neurons discriminated by complete CFSE staining (Fig. 8c). This decreased phagocytic ability in BV2Gal3KO cells was primarily attributed to the absence of GAL3, as the addition of exogenous GAL3 restored phagocytic activity to levels observed in BV2WT cells. These experiments underscore the critical role of GAL3 as an opsonin specifically under necrotic cell death condition triggered by the combined treatment of MPP^+^ and zVAD-fmk (overall caspase inhibition). Live-cell imaging analysis revealed that the effect of exogenous GAL3 was maximal during the early time points (first 3 h), after which its impact progressively declined (Suppl. Fig. 5a–c). Furthermore, live-cell imaging of phagocytosis revealed that cell contact is critical 30 to 60 min before the onset of phagocytosis (Figs. 8d, e and Suppl. Figure 5d). Thus, the analysis revealed that BV2 microglia first make cell contact with N27 neurons, after which the neurons are kept in contact with the BV2 microglia until neuronal death occurs. Subsequently, the neurons are either completely phagocytosed (Fig. 8e, and Suppl. Video 4), or phagocytosed in smaller particles (Fig. 8d, and Suppl. Video 3).

Overall, our in vivo and in vitro results demonstrate that necrosis of dopaminergic neurons triggers an MGnD-like phenotype in microglia characterized by a pronounced increase in phagocytic activity on necrotic and viable cells. We proved evidence supporting the crucial role of GAL3 as an opsonin in driving phagocytosis of diseased dopaminergic neurons.

## Discussion

Apoptosis has long been thought to have a critical role in neurodegeneration, and indeed, different studies have consistently reported a prominent role for both intrinsic and extrinsic apoptosis in the death of nigral dopaminergic neurons in PD [8, 60–62]. However, cell death backup mechanisms are especially relevant when apoptosis is blocked [63]. This view is important considering potential antiapoptotic therapies to combat neurodegenerative diseases. Therefore, it is necessary to dig into the fate of degenerating nigral dopaminergic neurons under selective genetic ablation of *Casp3*, the main effector caspase of the apoptotic pathway. We must note that in a previous study, Yamada et al. analysed the effect of non-specific caspase-3 gene disruption in an acute MPTP model [12]. They found significant protection of the nigrostriatal dopaminergic system and prevention of microglia activation. However, it is important to note that the acute MPTP model primarily induces non-apoptotic cell death [64, 65], which is accompanied by a strong microglial response in the ventral mesencephalon [66]. It should be taken in consideration, that as we have established, caspase-3 is playing a critical role in promoting proinflammatory microglial activation [66], which has been shown to be detrimental to the dopaminergic system [67]. To overcome these limitations, we used the subacute MPTP dose regimen, which has been shown to trigger apoptotic death of SN dopaminergic neurons due to inhibition of respiratory chain complex I [10, 11, 31, 68]. Complex I inhibition leads to mitochondrial permeabilisation (MOMP), which is considered the point-of-no-return of the apoptotic pathway. MOMP upstream interventions, i.e. Bax or p53 ablation, have demonstrated almost complete protection of the nigrostriatal dopaminergic system [10, 11]. However, in most cellular systems, interventions downstream of MOMP, i.e. caspase inhibitors, failed to prevent cell death [16]. With these precedents, we have developed a unique mouse strain that carried a hemizygous deletion for *Casp3* while the second allele was floxed to ensure an efficient deletion of *Casp3* in nigral dopaminergic neurons through Cre-recombinase under the control of the endogenous dopaminergic TH promoter (TH-C3KO) [69]. Our study was aimed at elucidating the molecular consequences of caspase-3 deletion in response to the MPTP challenge. We particularly focused on the brain immune response triggered by microglial cells, considering that apoptotic death represses undesirable activation of the immune system, including associated deleterious effects [17, 70]. Overall, selective gene ablation of *Casp3* failed to protect the nigral dopaminergic neurons after MPTP insult. Instead, nigral dopaminergic neurons switched cell death mode from apoptosis to necrosis along with strong activation of microglia that exhibited typical features of the microglial neurodegenerative phenotype (MGnD) [24]. Interestingly, we provide evidence that under dopaminergic caspase-3 deletion, microglia-derived GAL3 opsonises injured but apparently viable dopaminergic neurons to promote their phagocytosis [27, 71]. Finally, we did not find evidence of necroptosis in primary dopaminergic neurons in response to MPP^+^ challenge under concomitant caspase inhibition or in our mouse model in response to MPTP in conditions of *Casp3* ablation.

Since caspase-3 activation has long been known as a hallmark of apoptosis [42, 72], we first analysed this feature in response to MPTP challenge in WT and TH-C3KO mice. In line with previous studies [10, 11, 31, 68], our data clearly support the occurrence of apoptosis in WT/MPTP mice, relying on the presence of cleaved-caspase-3. Apoptosis was further confirmed by detection of cleaved-PARP, a caspase-3 activation product [43]. Importantly, none of these features were found in TH-C3KO/MPTP mice, indicating the effective ablation of apoptosis in these mice and supporting the relevance of our experimental design. Stereological analysis of nigral dopaminergic neurons and quantification of dopaminergic nerve terminals in striatal tissue demonstrated a high degree of selective loss of nigro-striatal dopaminergic cell bodies and terminals in WT/MPTP mice, which, however, was not reversed in TH-C3KO/MPTP. These observations confirm and extend previous studies showing that inhibition of caspase activation downstream of MOMP is ineffective in preventing cell death [17, 45, 73, 74].

The primary function of caspase-dependent apoptosis is the elimination of dead cells avoiding inflammation and associated deleterious effects [17, 18]. Consequently, we analysed the microglial response in TH-C3KO/MPTP and WT/MPTP animals. Our morphometric analysis revealed striking microglia activation in the ventral mesencephalon of TH-C3KO/MPTP mice, which was restricted to the SNpc and completely absent in WT/MPTP mice. This observation fully agrees with previous studies supporting an immunosuppressive function for caspase activation during intrinsic apoptosis [17, 75]. In fact, failure to activate caspases has been reported to change the fate of cell death, causing a switch from apoptosis to necrosis [45, 46], with the subsequent release of DAMPs [17]. The latest could explain the increased microglial response observed in TH-C3KO/MPTP mice. Membrane permeabilisation characterises necrotic cell death compared to apoptosis, and thus, we *premortem* injected PI to label the exposed DNA in our model. The presence of PI-labeled TH^+^ cell debris inside microglia cytoplasm in TH-C3KO/MPTP but not in WT/MPTP mice points out necrosis of dopaminergic neurons and subsequential phagocytosis by microglia cells.

Necroptosis is an orderly programmed cell death pathway that could play a role in our experimental scenario [76]. Similar to necrosis, necroptosis involves cell membrane permeabilisation, but in this case, activation of RIPK3 triggers phosphorylation of MLKL and its incorporation into the plasma membrane, ultimately leading to cell lysis [77]. In our model, we failed to detect evidence of necroptosis based on pMLKL detection in WT and TH-C3KO mice challenged with MPTP. The occurrence of necroptosis in the ventral midbrain in response to the MPTP challenge is also under debate. Two studies have suggested the possible occurrence of necroptosis in nigral dopaminergic neurons following subacute MPTP regime [48, 49]. In one of these studies, the administration of RIPK1 inhibitor Nec-1 conferred partial protection against MPTP-induced damage of the nigrostriatal dopaminergic system [48]. In a second study, pMLKL was detected in nigral dopaminergic neurons in response to MPTP treatment [49]. Our data demonstrating the absence of MLKL activation (pMLKL) and lack of significant microglia activation in WT mice argue against necroptosis as a prominent cell death mode after MPTP administration. As previously mentioned, we did not detect pMLKL in the TH-C3KO/MPTP animal group, although in this case, we recognised evident signs of microglial activation. In this line, in a third study, the deletion of RIPK3 protected from dopaminergic neurodegeneration in the ventral mesencephalon in the acute MPTP mouse model, but in this case, associated with intrinsic apoptosis rather than necroptosis [50]. Importantly, in this last referred study, the authors were unable to find signs of microglia activation neither in the striatum nor in the ventral midbrain, thus mimicking our results, which argues against the occurrence of necroptosis in the nigrostriatal system. Indeed, necroptosis remained undetected under these conditions, and treatment with Nec-1s failed to confer protection on the nigral dopaminergic system [50]. To elucidate the potential contribution of necroptosis to the fate of dopaminergic neurons, we performed mesencephalic neuron primary cultures that were exposed to MPP^+^ combined with pan-caspase/caspase-8 inhibition (zVAD-fmk and IETD-fmk) and RIPK1 inhibition (Nec-1). Caspase-8 is the most critical molecule that negatively regulates necroptosis by proteolytic cleavage of RIPK1, an event that prevents the association between RIPK1 and RIPK3, thus preventing the necrosome assembly [78]. Thus, it is well established that caspase-8 inactivation is a prerequisite for susceptibility to necroptosis in most cell types [30, 79]. Our in vitro study clearly demonstrates that following MPP^+^ challenge: i) dopaminergic neurons exhibit typical features of apoptosis; ii) pan-caspase/caspase-8 inhibition does not confer protection on nigral dopaminergic neurons and, besides, promotes a switch from apoptosis to necrosis, a view already seen by Hirsch and colleagues [7]; iii) promotion of the necrotic death was more deleterious for dopaminergic neurons as deduced from quantification of neurite length; and iv) the switch from apoptosis to necrosis was not associated with necroptosis as Nec-1 failed to confer protection against the concomitant treatment of MPP^+^ and caspase inhibition. Overall, we conclude that caspase inhibition does not provide neuroprotection for dopaminergic neurons condemned to apoptotic death. Instead, the fate of neuronal death changes from apoptosis to necrosis in both in vivo and in vitro models. Moreover, in our in vitro model, this switch translates into reduced neurite extension, an event related to reduced neuronal survival [80].

As previously mentioned, a notable characteristic observed in TH-C3KO/MPTP but not in WT/MPTP mice was an intensified microglia response in the ventral mesencephalon. Under disease conditions, microglia may switch from a homeostatic state to a DAM/MGnD phenotype, which is characterised by upregulation of genes highly involved in phagocytosis such as *Trem2, Itgax*, *Spp1*, *Clec7a*, and *Lgals3* [23, 24]. With these precedents, we next wondered if microglia acquire a DAM/MGnD phenotype in WT and TH-C3KO mice challenged with MPTP. Hence, we first measured selective markers of MGnD microglia, including *Trem2*, *Lgals3*, *Clec7a*, and *Cd68* by qPCR. All these markers were upregulated in WT/MPTP and highly exacerbated in TH-C3KO/MPTP mice. In fact, immunofluorescence of TREM2 and GAL3 evidenced a dramatic difference between WT and TH-C3KO mice after the MPTP challenge, as a subset of microglia expressing high levels of TREM2 and GAL3 was precisely confined to the SNpc exclusively in the transgenic mice. Overall, we can conclude that selective ablation of caspase-3 in dopaminergic neurons highly promotes the MGnD phenotype in the ventral mesencephalon in response to MPTP. Remarkably, our immunofluorescence study revealed a specific subtype of GAL3-expressing microglia displaying phagocytic cups engulfing apparently viable dopaminergic neurons. Importantly, we and others have demonstrated that GAL3 is released by activated microglia [27, 81, 82] and, indeed, a diffuse GAL3 staining was evident in dopaminergic neurons contacting GAL3 highly expressing microglia. 3D reconstruction from confocal images was used to confirm that GAL3 labelling is detected on the membrane surface of dopaminergic neurons in close contact with microglia. These findings reinforce the notion that GAL3 opsonises viable dopaminergic neurons injured by MPTP treatment to promote their phagocytosis. Supporting this, CD68, a type I transmembrane glycoprotein mainly associated with the endosomal/lysosomal compartment [83, 84], was found to be acutely up-regulated in the midbrain from TH-C3KO/MPTP. This lysosomal marker co-localized with GAL3 and TH, indicating an increased microglial phagocytic activity, further bracing the idea that GAL3 may act as an opsonin by binding to N-acetyl-lactosamine motifs exposed on the membrane surface of injured neurons [27, 28]. Previously, we observed that exogenous GAL3 enhances the phagocytic capacity of BV2 cells and that GAL3-expressing microglia is associated with degenerating hippocampal neurons in a model of traumatic brain injury along with its release into the cerebrospinal fluid [85]. Additionally, we and others have demonstrated the ability of GAL3 to activate important receptors associated with microglia phagocytosis, including TREM2 [81] and MERTK [27], and TLR4 [82, 85], which is associated with pro-inflammatory microglia activation. This raises the question of whether GAL3 plays a significant role in mediating the phagocytosis of injured but viable dopaminergic neurons, a process known as primary phagocytosis or phagoptosis, as recently reviewed by Guy Brown [59]. The role of GAL3 as an opsonin has been demonstrated in various scenarios [86, 87], but not, to our knowledge, in PD context. Since phagoptosis and phagocytosis involve “eat-me signals” exposed or released by stressed or dying neurons [88], we developed an in vitro setup combining BV2 cells lacking GAL3, injured N27 dopaminergic cells and exogenous GAL3. Our results showed that both endogenous and exogenous GAL3 significantly promote the phagocytosis of dopaminergic neurons when subjected to a combination of MPP^+^ and zVAD-fmk (previously shown to mainly induce necrosis), but not to MPP^+^ alone (predominantly inducing apoptosis). Furthermore, our live-cell imaging analysis revealed extensive microglia-neuron contact, followed by neuronal death before phagocytosis. These observations, consistent with our in vivo findings, underscore the potential relevance of GAL3 as an “eat-me signal”.

In summary, we provide evidence that the interruption of the apoptotic program within dopaminergic neurons, beyond redirecting cell death mode from apoptosis to necrosis, highly enhances microglia activation acquiring a MGnD-like phenotype. Notably, this microglia subpopulation exhibited a remarkable phagocytic activity towards both dead and apparently viable dopaminergic neurons following a GAL3-dependent mechanism. Enhanced microglial phagocytic activity is potentially harmful, as it may eliminate healthy synapses [89] and increase neuronal cell loss contributing to neurodegeneration [86]. Our study does not support caspase inhibition as a therapeutic option for PD; however, pharmacological targeting of GAL3 appears to be a promising preclinical strategy to combat exacerbated deleterious microglial response towards the dopaminergic system in neurodegenerative conditions. Indeed, we have recently reported the detrimental impact of GAL3 on the aggregation process of α-synuclein and the formation of Lewy bodies, which are key pathological features observed in PD [39]. Furthermore, recent GWAS studies have indicated a causal relationship between GAL3 and PD, as single nucleotide polymorphisms found in the human *LGALS3* gene have been linked to a higher risk of developing the PD condition [90]. This critical insight sheds new light on the role of GAL3 in the progression of PD.

### Supplementary information

Supplementary Figures and Legends

Supplementary Video 1

Supplementary Video 2

Supplementary Video 3

Supplementary Video 4

## Data Availability

All data generated or analysed during this study are included in this published article [and its supplementary information files].
