# Supplementary material for: Dopaminergic neurons lacking Caspase-3 avoid apoptosis but undergo necrosis after MPTP treatment inducing a Galectin-3-dependent selective microglial phagocytic response
Source: Cell Death Dis. 2024 Aug 27;15(8):625. doi: 10.1038/s41419-024-07014-9 (PMC11369297; doi:10.1038/s41419-024-07014-9)

Supplementary Figure 1. Effect of MPTP on PARP cleavage in dopaminergic neurons of SNpc of WT and TH-C3KO animals. *a*, Coronal sections of SNpc immunostained for TH and Cleaved-PARP from WT and TH-C3KO animals injected with saline solution or MPTP and analyzed hours after the third injection. Yellow dashed lines highlight dopaminergic neurons in SNpc expressing Cl-PARP in WT/MPTP condition. Scale bar, 30 µm. *b*, Higher magnification area of WT/MPTP condition in *a* enclosed by red dashed line with arrows pointing dopaminergic neurons expressing Cl-PARP. Scale bar, 10 µm.

Supplementary Figure 2. Effect of MPTP on the dopaminergic striatal innervation of WT and TH-C3KO animals. *a*, Coronal sections of striatum immunostained for TH of WT and TH-C3KO animals injected with saline solution or MPTP and analyzed 4 or 28 days after the last injection. Scale bar, 300 µm. *b,* Densitometric analysis of TH immunostained striatum. *c,* Quantification by HPLC of dopamine in striatum. Data represent the mean ± SEM from at least three animals. Statistical analysis: One-way ANOVA followed by the Fisher's LSD *post hoc* test for multiple comparisons was used, with $\alpha=0.05$. In panel *b*, (a), compared with the WT/saline group; (b), compared with the TH-C3KO/saline group; (c) compared with the WT/MPTP d4 group; (d), compared with the TH-C3KO/MPTP d4 group; (e), compared with the WT/MPTP d28 group. In panel *c*, (a), compared with the WT/saline group; (b), compared with the TH-C3KO/saline group; in panel *b*, $p < 0.05$; in panel *c*, $p< 0.001$.

Supplementary Figure 3. Effect of MPTP on the microglia activation status of SNpc and striatum of WT and TH-C3KO animals. *a*, Quantitative assessment of different microglial activation states in SNpc expressed as relative percentage based on morphological criteria and IBA-1 expression in immunostained sections. Representative examples are shown depicting transition of homeostatic to activated state with three morphological types such as homeostatic, hypertrophic and ameboid-like. Scale bar, 15 µm. *b*, Quantitative assessment of different microglial activation states in striatum based on morphological criteria expressed as relative percentage. The same assignment of activation states based on morphological criteria was applied as in SNpc. *c*, Quantification of IBA-1^+ cells/mm^2 in immunostained striatum sections of WT and TH-C3KO animals injected with saline solution or MPTP and analyzed 4 days after the last injection. Data represent the mean ± SEM from at least three animals. Statistical analysis: One-way ANOVA followed by the Fisher's LSD *post hoc* test for multiple comparisons was used,

with α=0.05. In *a*, (a), compared with the WT/saline group; (b), compared with the TH-C3KO/saline group; (c) compared with the WT/MPTP. In panel *a*, $p < 0.01$, in panel *b*, $p < 0.05$.

Supplementary Figure 4. Phosphatidylserine is exposed in stressed but viable neurons in a caspase-independent manner. *a*, N27 and BV2 cells co-culture (as shown in Fig. 8a) were stained for Annexin-V-APC to detect phosphatidylserine exposure. Surviving attached N27 neurons presented normal cell and nuclei morphology with no signs of apoptosis. White arrows indicate viable Annexin-V negative neurons. Yellow arrows indicate viable Annexin-V positive neurons as a sign of cellular stress. Scale bar 10 µm. *b*, Annexin-V mean fluorescence intensity (MFI) was measured exclusively in the membrane of N27 neuronal cells. The cell membrane was identified as the border of CFSE staining enlarged for 3 pixels in all directions. Data represent the mean ± SEM, n=4. Statistical analysis: One-way ANOVA followed by the Fisher's LSD *post hoc* test for multiple comparisons was used for individual and group comparisons. with α=0.05. **** represents $p < 0.0001$.

Supplementary Figure 5. Live cell phagocytosis assay reveals a time-dependent effect of exogenous GAL3. *a*, Live cell tracking of phagocytosis for 6 hours in Operetta CLS High-Content Analysis System. BV2 cells were co-cultured with prestained N27 cells in control situation and treated with MPP^+zVAD-fmk. The percentage of BV2 cells with any fragment of N27 cell in their cytoplasm is plotted. Two different stages were identified based on the results observed for the BV2Gal3KO+GAL3 MPP^+zVAD-fmk group: the first three hours were identified to exhibit an effect for exogenous GAL3 promoting phagocytosis (shown in green and results plotted in *b)*; the rest of the assay showed no effect for exogenous GAL3 (shown in pink and results plotted in *c)*. Data represent the mean of 3-4 experiments. *b*, Area under the curve of the first three hours of the assay is measured; n≥3. Data represent the mean ± SEM. *c*, Area under the curve of the last three hours of the assay is measured; n≥3. Data represent the mean ± SEM. *d*, Example of extreme cell contact, tropism and phagocytosis observed during live-cell tracking. N27 neurons are stained with CFSE (in green), cell cytoplasm is detected by Mitotracker (in red), and nuclei are stained with Hoechst 33258 (in blue). Scale bar 20 µm. Statistical analysis: One-way ANOVA followed by the Fisher's LSD *post hoc* test

for multiple comparisons was used, with $\alpha=0.05$. *** represents $p < 0.001$;** represents $p < 0.01$; * represents $p < 0.05$.

Supplementary Video 1. Video of Imaris 3D reconstruction from the image represented in Fig. 3b.

Supplementary Video 2. Video of the image represented in Fig. 7e.

Supplementary Video 3. Video from live cell tracking represented in Fig. 8d.

Supplementary Video 4. Video from live cell tracking represented on Fig. 8e.

SUPPL. FIGURE 1

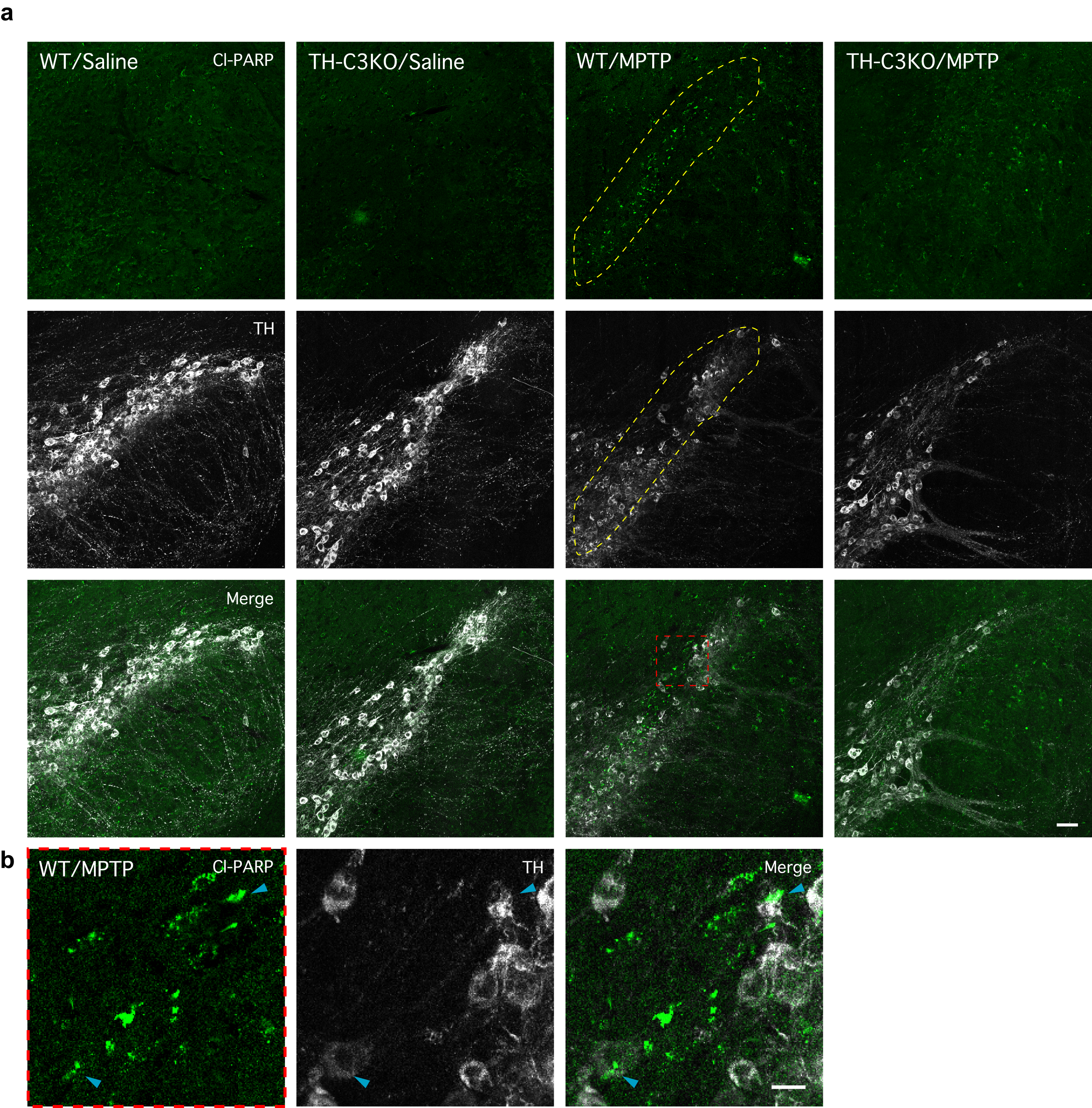

SUPPL. FIGURE 2

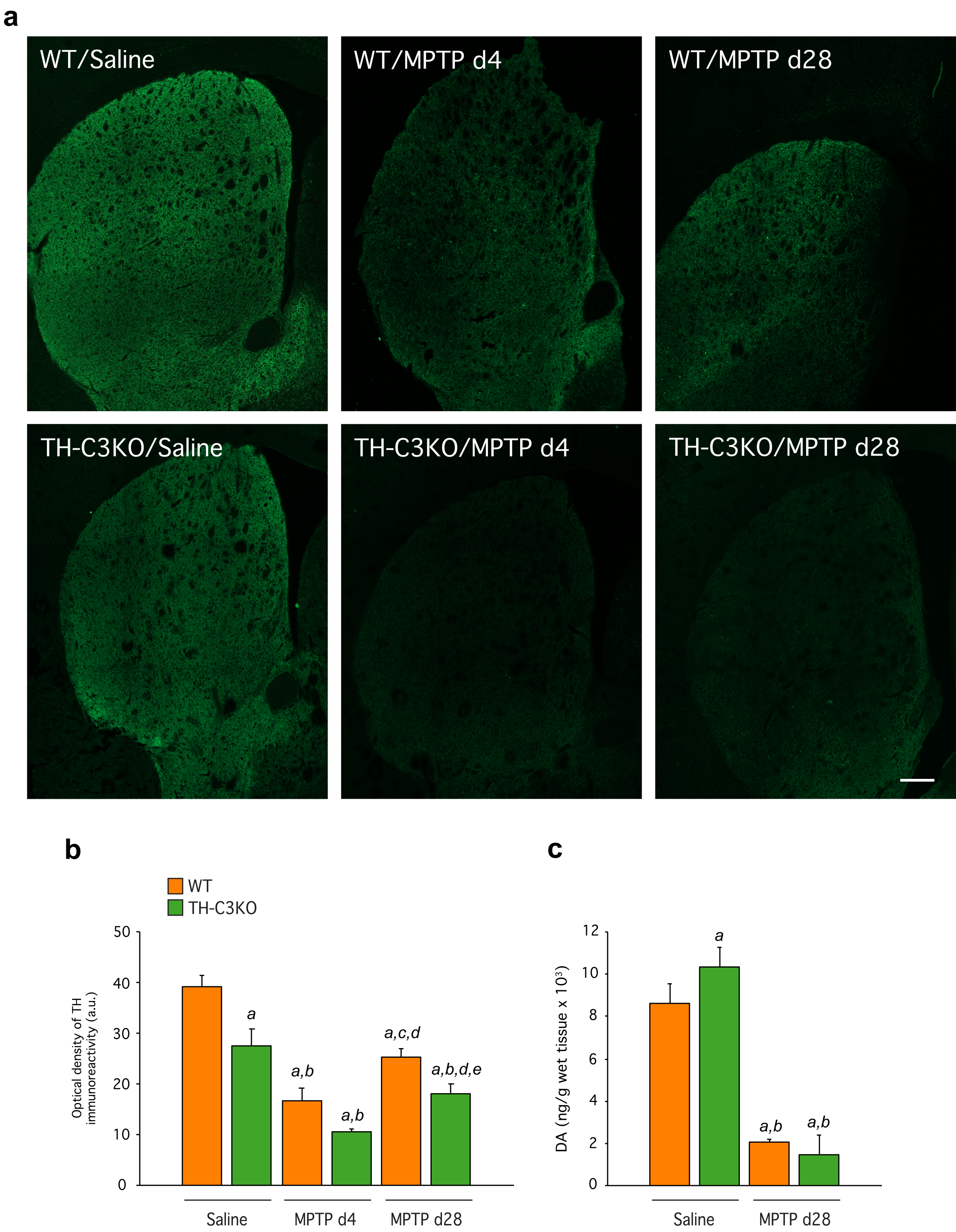

SUPPL. FIGURE 3

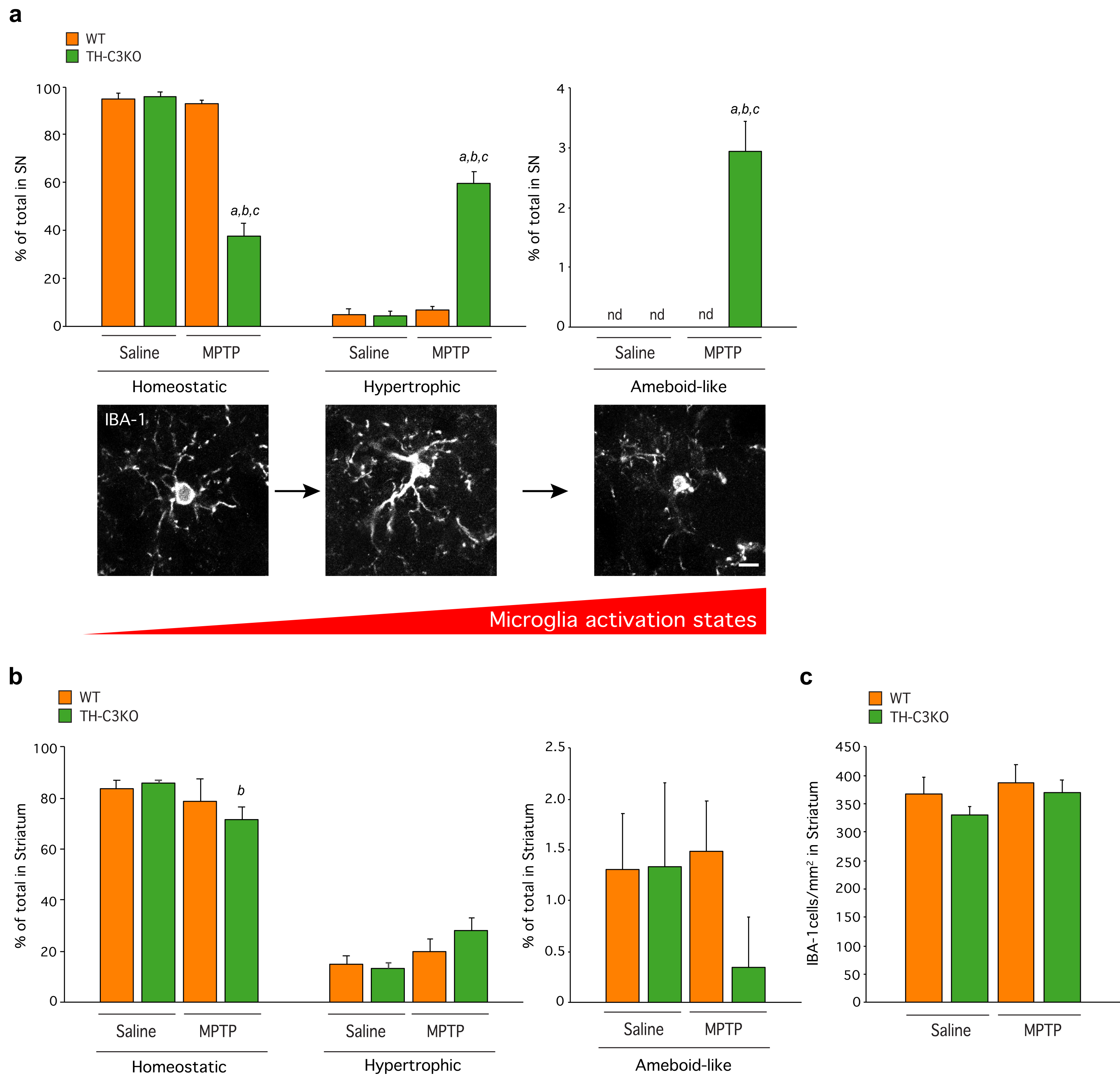

SUPPL. FIGURE 4

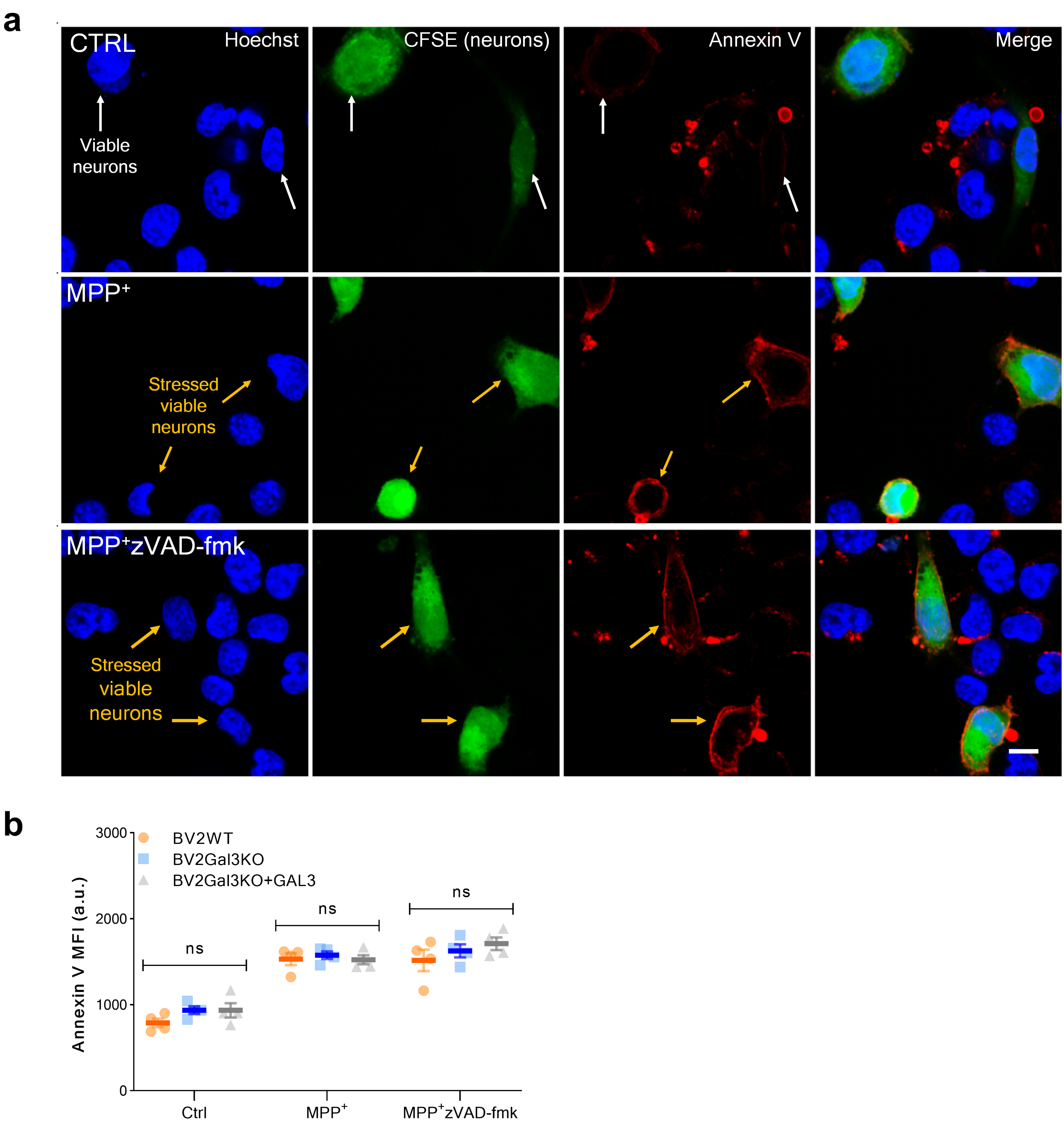

Ctrl vs. MPP^+	****	<0.0001
Ctrl vs. MPP^+zVAD-fmk	****	<0.0001
MPP^+ vs. MPP^+zVAD-fmk	ns	0.2246

SUPPL. FIGURE 5

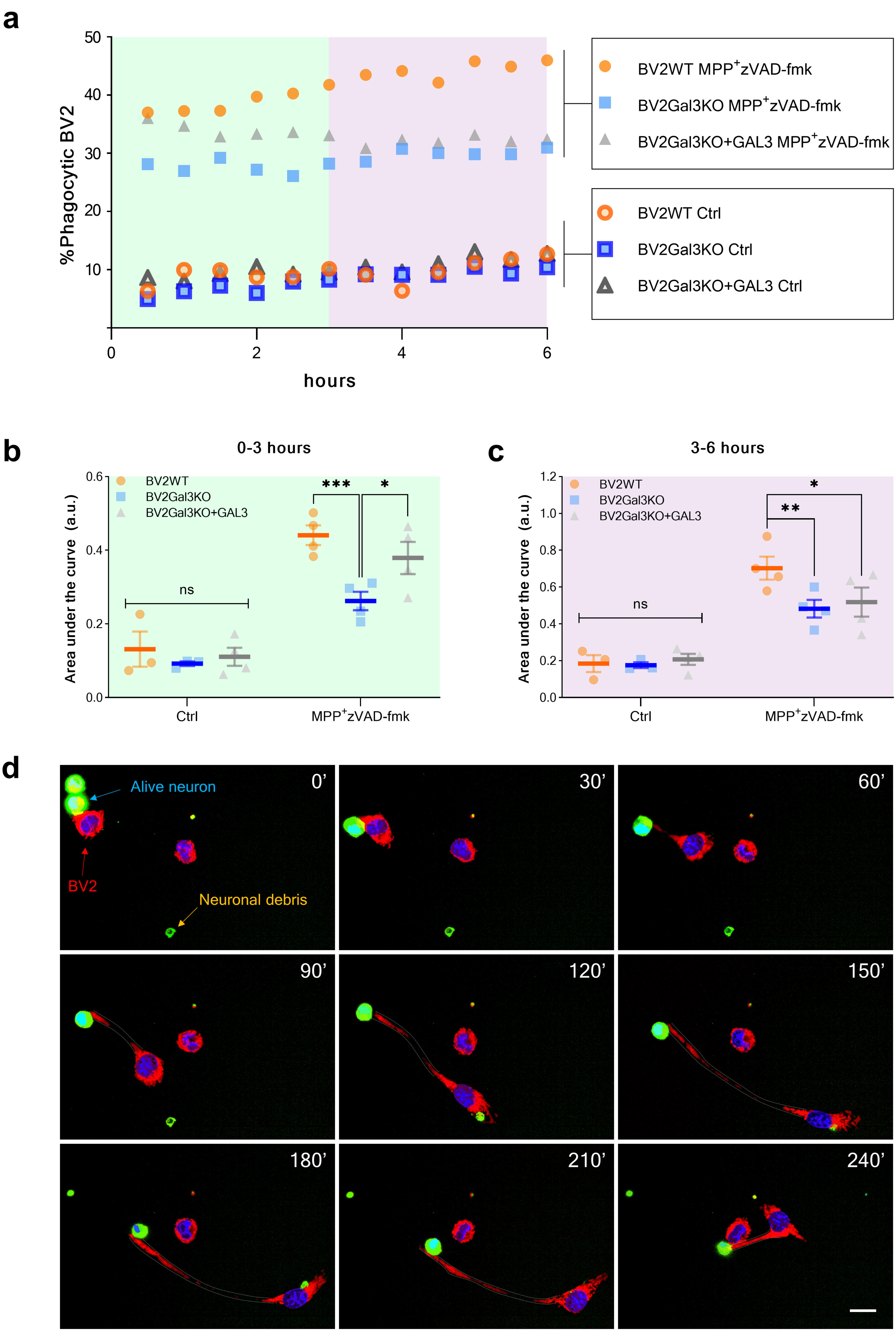

Supplement: Supplementary file 1 — Supplementary Figures and Legends [file 41419_2024_7014_MOESM1_ESM.pdf]
